# Cross-species functional analysis of a *de novo DCLK1* variant associated with a neurodevelopmental disorder

**DOI:** 10.21203/rs.3.rs-10425123/v1

**Published:** 2026-07-23

**Authors:** David F. Butler, Weimin Yuan, Hirokazu Hashimoto, Hieu Hoang, Jonathan H. Sheehan, Vanessa Gomez, Jill A. Rosenfeld, Lindsay C. Burrage, Gary A. Silverman, Patricia Dickson, Karen K. Moeller, Lisa Emrick, Mythily Ganapathi, Undiagnosed Diseases Network, Dustin Baldridge, Oguz Kanca, Carlos Bacino, Brendan Lee, Hugo J. Bellen, Shinya Yamamoto, Wendy K. Chung, Tim Schedl, Michael Wangler, Andrew Yoo, Stephen C. Pak

**Affiliations:** 1 Department of Pediatrics, Washington University School of Medicine, St Louis, MO 63110, USA; 2 Department of Molecular & Human Genetics, Baylor College of Medicine, Houston, TX 77030, USA; 3 Jan and Dan Duncan Neurological Research Institute, Texas Children’s Hospital, Houston, TX 77030, USA; 4 Department of Medicine, Washington University School of Medicine, St Louis, MO 63110, USA; 5 Texas Children’s Hospital, Houston, TX 77030, USA; 6 Department of Radiology, Baylor College of Medicine, Houston, TX 77030, USA; 7 Department of Pathology and Cell Biology, Columbia University Irving Medical Center, New York, NY 10032, USA; 8 Department of Pediatrics, Boston Children’s Hospital, Harvard Medical School, Boston, MA 02115 USA; 9 Department of Genetics, Washington University School of Medicine, St Louis, MO, 63110 USA; 10 Department of Developmental Biology, Washington University School of Medicine, St Louis, MO 63110, USA

**Keywords:** DCLK1, neurodevelopmental disorder, undiagnosed diseases, functional genomics, *Caenorhabditis elegans*, patient-derived neurons, neurite degeneration

## Abstract

Neurodevelopmental disorders are genetically heterogeneous and often remain unresolved despite extensive clinical evaluation and genomic testing. Here, we report a proband with a progressive neurodevelopmental disorder evaluated through the Undiagnosed Diseases Network who harbored heterozygous *de novo* missense variants in two genes, *DCLK1* (p.(S228L)) and *SFPQ* (p.(P623R)). To determine the clinical significance of these candidate variants, we employed an integrative pipeline combining structural modeling, cross-species functional genomics, and patient-derived neuronal analyses. While the *SFPQ* variant yielded no detectable phenotype in *Drosophila melanogaster*, modeling the *DCLK1* p.S228L variant in *Caenorhabditis elegans* induced severe locomotor deficits and aberrant neuronal morphology, including neurite blebbing. Parallel analyses of directly reprogrammed patient-derived neurons recapitulated these neurite defects, characterized by neurite beading, swelling and fragmentation, and elevated apoptosis. Transcriptomic profiling revealed dysregulation of neurodevelopmental and axon-guidance pathways alongside molecular signatures of neurodegeneration. Crucially, exogenous expression of wild-type *DCLK1* or pharmacological targeting of a downstream dysregulated pathway partially rescued the neurite defects. Collectively, our findings implicate *DCLK1* in a previously unrecognized progressive neurodevelopmental disorder and demonstrate the power of integrative cross-species functional genomics in resolving ultra-rare disease variants.

## INTRODUCTION

The Undiagnosed Diseases Network (UDN) was established to accelerate the diagnosis of rare and previously unrecognized diseases by integrating cross-disciplinary expertise, advanced genomic technologies, and standardized evaluation protocols (Ramoni et al., 2017). Patients referred to the UDN frequently present with rare, novel, or atypical manifestations of disease that complicate clinical recognition and often result in prolonged diagnostic odysseys. These challenges are particularly pronounced in n-of-1 cases, in which only a single individual with a candidate gene variant and associated phenotype has been identified. To address these complexities, the UDN promotes collaborative expert review, extensive data sharing, and rigorous analytical frameworks that bridge clinical care and translational research. The UDN’s evaluation process incorporates deep phenotyping, genomic sequencing, RNA sequencing, metabolomics, and functional studies in model organisms (Baldridge et al., 2021; Furuta et al., 2024; Zhao et al., 2025).

Analysis of genomic sequencing data in unresolved cases frequently identifies more than one potentially relevant variant contributing to the proband’s phenotype (Kobren et al., 2025; Smith et al., 2019). In addition, dual or multiple candidate gene variants have been reported in approximately 2–5% of cases evaluated by clinical diagnostic laboratories (Karaca et al., 2018; Posey et al., 2017). In these situations, candidate variants often require parallel investigation to define their relative contribution to disease. The diagnostic challenge becomes even greater when implicated genes have not previously been associated with human disease, necessitating extensive functional characterization using complementary approaches that may include model organisms, patient-derived cell systems, and *in vitro* assays.

Here, we describe an individual with a history of unexplained developmental disorder evaluated through the UDN who harbors heterozygous *de novo* variants in two genes of uncertain clinical significance, *DCLK1* p.(S228L) and *SFPQ* p.(P623R). Both gene variants emerged as strong candidates based on their pathogenicity predictions and predicted involvement in neurological functions. To investigate the pathogenic relevance of these variants, both genes were submitted to the UDN Model Organism Screening Center (MOSC) (Baldridge et al., 2021) for experimental studies in *C. elegans* (*DCLK1*) and *Drosophila* (*SFPQ*) and, based on these findings, were followed by studies with directly reprogrammed patient neurons. Collectively, these studies provide functional evidence that the *DCLK1* p.(S228L) variant is the primary driver of the proband’s clinical phenotype and establish *DCLK1* as a candidate gene for a previously unrecognized neurodevelopmental disorder.

## RESULTS

### Case study:

The patient is a 10-year-old male who presented with unexplained developmental regression starting at age 3. At 3 years of age, the patient was noted to have an initially subtle lack of progression in gross motor abilities and began occupational therapy shortly thereafter. At age 4, the patient began to struggle in a traditional school setting, with anxiety and emotional outbursts, and was pulled out of school during that time. Concurrently, the patient’s speech ability began to gradually decline, and he was referred to neurology and developmental pediatrics. He was diagnosed with autism spectrum disorder at 5 years of age, with his speech continuing to decline, but he was still able to respond to questions and make requests with a single word. The patient then started Applied Behavior Analysis (ABA) therapy full time and experienced significant developmental regression during that time. His speech significantly declined, and he began losing other basic life skills, like inability to get dressed without assistance or being able to lock/unlock the door. At 7 years of age, the patient became completely non-verbal, developed insomnia, and lost many of the skills he previously developed, like using utensils and toilet training. A 24-hour continuous video EEG testing was done at age 7 to observe if regression was due to seizure activity during sleep. The EEG was found to be abnormal with an increased spike wave index during sleep, but the spike wave index (SWI) was not high enough to meet the diagnosis criteria of electrical status epilepticus in sleep, and no clinical seizures were noted. However, at age 8, continuous video EEG testing was repeated, and results confirmed the patient had epilepsy with electrical status epilepticus in sleep (ESES) with focal impairment awareness seizures. The EEG captured 3 left temporal seizures that were unrecognized clinically. During two of the seizures the patient stared off and had right hand automatisms. According to the parents, it was hard to know when seizures start and how often they occur, as they are not associated with clinically recognizable change in behavior. The EEG also showed multifocal and generalized epileptiform discharges and slowing. At present, seizures appear to be well controlled with oral steroids and Clobazam. Brain Magnetic Resonance Imaging (MRI) was done at ages 4, 7, and 10 years (Supplemental Figure S1). Brain MRIs at age 4 and 7 were interpreted as normal. However, at age 10 there were subtle findings in the right temporal lobe, suggestive of possible focal cortical dysplasia versus nonspecific gliosis (Supplemental Figure S1). The patient was seen by genetics at 7 years old, and trio exome sequencing (ES) revealed a paternally inherited *CACNA1A* p.(D1723G) variant. The father does not have neurobehavioral symptoms, so this variant was considered not to contribute to the patient’s symptoms. Because the condition remained unexplained, the patient was referred to the Undiagnosed Diseases Network (UDN) at the age of 8 years in the hopes of discovering an underlying genetic cause for the patient’s clinical presentation (Supplemental Figure S2).

### Variant bioinformatics:

Research reanalysis of the clinical trio ES data identified two candidate *de novo* missense variants with damaging bioinformatics predictions, both in genes not currently associated with human disease but with pLI (probability of loss-of-function intolerance) scores of 1 (Supplemental Table S1) (Lek et al., 2016). The first gene was in *DCLK1* (doublecortin-like kinase 1, NM_001330071.2:c.683C>T, p.(S228L), which had strong pathogenicity predictions (Supplemental Table S1) (Ioannidis et al., 2016). The variant was absent in earlier versions of gnomAD, although it is present one time in version 4.1.1 (including UK biobank) (Chen et al., 2024). The S228 residue is conserved in mouse to *C. elegans* while *Drosophila* has a conservative threonine substitution (MARRVEL Beta v2.0) (Wang et al., 2017) suggesting that this residue may be important for *DCLK1* function. This variant was considered to be a candidate for causing the patient’s neurologic features due to the gene’s suspected role in both neuronal migration and in nervous system function. Mice homozygous for a null *Dclk1* allele were viable and fertile but showed abnormalities in the corpus callosum and hippocampal commissure (Koizumi et al., 2006). The second candidate variant was in *SFPQ* (splicing factor, proline and glutamine rich, NM_005066.3:c.1868C>G, p.(P623R)), which also had strong pathogenicity prediction scores (Supplemental Table S1). The variant is absent in gnomAD v4.1.1 (including UK biobank), although p.(P623H) and p.(P623S) are present 13 and 2 times, respectively. Homozygous *Sfpq*-knockout mice are lethal (Kowalska et al., 2012), however, conditional knockout in the brain results in widespread alterations of long-gene transcripts, and the gene is linked to neurodegenerative disease (Lim et al., 2020; Takeuchi et al., 2018).

### Genomic matchmaking:

In an attempt to gather further evidence for possible pathogenicity of variants of either candidate gene, GeneMatcher (Sobreira et al., 2015) entries were created for the genes. For the *DCLK1* matches, these were not pursued further due to one or more of the following reasons: 1) incomplete information regarding variant inheritance pattern, 2) lack of phenotypic overlap with our case and others, in particular regression and epilepsy were not reported for any other cases, or 3) other genes or genetic conditions were being considered as possibly diagnostic.

Via GeneMatcher, we also connected with a group gathering data on patients with mostly *de novo SFPQ* missense heterozygous variants, with neurodevelopmental phenotypes with varying severity and additional symptoms. Regression had not been noted in their cohort, although there were cases with epilepsy or abnormal EEGs. Given the absence of specific phenotypic overlap with the regression and ESES phenotypes for other patients with variants in either gene, patient matchmaking efforts did not help to determine which candidate variant might be contributing to the patient’s phenotype.

### *SFPQ* functional studies in *Drosophila*

*SFPQ* (splicing factor proline and glutamine rich) encodes a multifunctional RNA- and DNA-binding protein that is involved RNA splicing, DNA repair, transcriptional regulation, paraspeckle formation and nuclear organization with important roles in neuronal development and maintenance (Fox & Lamond, 2010; Lim et al., 2020; Thomas-Jinu et al., 2017; Yu et al., 2024). *SFPQ* is evolutionarily conserved across vertebrates and invertebrates, including the fly *Drosophila melanogaster*. Two fly genes, *nonA* (*no on or off transient A*) and *nonA-l* (*nonA-like*), appear to be orthologous to human *SFPQ* as well as its paralogs *NONO* and *PSPC1* (Knott et al., 2016). To test whether the p.(P623R) variant in *SFPQ* causes any functional alteration, we took an overexpression approach since overexpression of the fly *nonA* has been shown to induce scorable phenotypes such as rough eye, and this assay has been used to study rare disease-associated human variants in *NONO* (Itai et al., 2023). We generated transgenic flies expressing reference or p.(P623R) variant human *SFPQ* using the UAS/GAL4 binary system. We examined the effects of both ubiquitous expression [*daughterless(da)-Gal4*] and tissue-specific expression using three drivers: *nubbin(nub)-Gal4* (wing), *pnr-Gal4* (dorsocentral thorax and other tissues), and *GMR-Gal4* (eye). Overexpression of the reference *SFPQ* transgene resulted in lethality using multiple drivers and a rough-eye phenotype across multiple temperatures (Supplemental Figure S3A). The p.(P623R) variant produced an indistinguishable phenotype under all drivers tested compared to the reference *SFPQ* (Supplemental Figure S3A). Western blot analysis of whole-fly lysates demonstrated comparable expression levels between the reference and variant SFPQ proteins (Supplemental Figure S3B). These results suggest that the p.(P623R) variant in *SFPQ* does not seem to have strong functional consequences, at least in the phenotypic contexts we examined, as well as on protein stability.

### DCLK1 protein structure modeling

Doublecortin-like kinase 1 (*DCLK1*) is a member of the doublecortin family, an evolutionarily conserved group of microtubule-associated proteins (MAPs) that regulate cytoskeletal organization and neuronal development (Lin et al., 2000; Liu et al., 2012; Molkentin et al., 1998; Ono et al., 1999; Ruggiero-Lopez et al., 1999; Shu et al., 2006). Previous animal studies indicated that *DCLK1* has enriched expression in the developing brain and plays a critical role in axonogenesis, dendritic growth, microtubule-associated vesicle transport, neurotransmission and cortical and hippocampal development (Deuel et al., 2006; Koizumi et al., 2017; Koizumi et al., 2006; Lipka et al., 2016; Murphy et al., 2023; Tanaka et al., 2006; Vreugdenhil et al., 2007; Yu et al., 2000). Variants in doublecortin family genes are associated with a spectrum of neurodevelopmental and sensory disorders, including X-linked lissencephaly and double-cortex syndrome (*DCX*) (des Portes, Francis, et al., 1998; des Portes, Pinard, et al., 1998; Gleeson et al., 1998; Leger et al., 2008), inherited retinal degeneration (*RP1*) (Bowne et al., 1999; Sullivan et al., 1999), and dyslexia (*DCDC2*) (Meng et al., 2005). Genome-wide association studies implicate single nucleotide polymorphisms in *DCLK1* in neuropsychiatric conditions such as schizophrenia, bipolar disorder, and attention deficit/hyperactivity disorder (Håvik et al., 2012); however, a definitive monogenic disease association for *DCLK1* has not yet been established.

*DCLK1*, together with doublecortin-like kinase 2 (*DCLK2*), represents a unique subfamily distinguished by the presence of a C-terminal serine/threonine protein kinase domain in addition to the N-terminal tandem DCX domains (Burgess et al., 1999; Kim et al., 2003). The kinase domain contributes to phosphorylation-dependent signaling in a context-dependent manner, including roles in axon elongation via phosphorylation of substrates such as MAP7D1 (Koizumi et al., 2017). In contrast, several core neuronal functions of DCLK1, including neuronal growth and intracellular cargo transport, are largely independent of kinase activity and are mediated by its microtubule-binding DCX domains (Koizumi et al., 2006; Lipka et al., 2016). Furthermore, the DCLK1 DCX domains can bind at least one MAP (e.g. a TACC family ortholog), independent of the DCX domain’s ability to bind microtubules (Bellanger et al., 2007).

**Figure 1.**
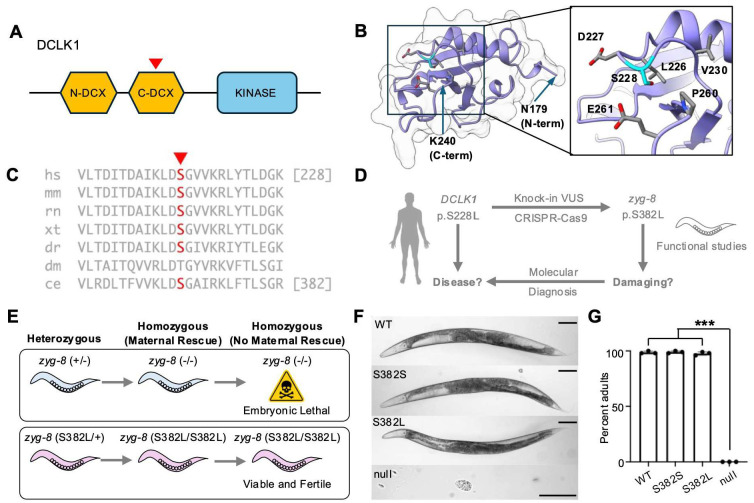
Protein modeling and functional studies in *C. elegans*. (A) Schematic of DCLK1 protein showing two N-terminal DCX domains followed by a C-terminal kinase domain. (B) DCLK1 variant S228L is predicted to stabilize the C-terminal doublecortin, C-DCX, domain. AlphaFold model AF-O15075-F1 is shown with transparent protein surface to illustrate the structural context of the variant. Position 228 is highlighted in cyan, and the sidechains of residues within 5.0Å are also shown. N179 and K240 are the N- and C-terminal residues of this domain. The ddG calculation predicts that replacing the polar serine sidechain at position S228 with a hydrophobic leucine sidechain will stabilize the structure by 4.1 Rosetta Energy Units, consistent with improving the intra-residue contacts in that region, and eliminating the buried polar oxygen. (C) C-DCX domain is conserved through evolution. Multiple sequence alignment of the region surrounding the variant in the C-DCX domain from human (hs), mouse (mm), rat (rn), frog (xt), fish (dr), fly (dm) and *C. elegans* (ce) is shown. The location of the proband’s variant, S228, is indicated by a red arrow and corresponds to S382 in *C. elegans*. (D) Schematic of the variant modeling approach in the *C. elegans*. (E) *zyg-8(−/−)* null mutants are maternal effect embryonic lethal. *zyg-8(S382L)* patient variant homozygous animals are viable and fertile. (F) Differential Interference Contrast (DIC) images of *C. elegans* 72 hours post egg-lay. (G) Quantification of percent adults 72 hours post egg-lay. Data from 3 biological replicates. n=100 (eggs) per genotype. Error bars indicate the mean with standard deviation. Differences between groups were determined using Kruskal-Wallis test followed by Dunn’s multiple comparison test. ns, not significant; a P-value <0.05 was considered statistically significant. *** indicates p value < 0.001.

In DCLK1, the tandem DCX domains (N-DCX and C-DCX) are structurally similar but divergent in sequence (Rufer et al., 2018). The proband’s affected residue, S228, is located in the C-terminal DCX domain (C-DCX) (Figure 1A). To assess the functional consequences of the S228L substitution on DCLK1 protein conformation and stability, we performed a Rosetta energetic analysis using the cartesian ddG protocol (Frenz et al., 2020; Park et al., 2016). The predicted AlphaFold structure of the C-DCX domain is shown in Figure 1B. Interestingly, the ddG calculations predict that replacing the polar serine sidechain with a hydrophobic leucine sidechain will stabilize the structure by 4.1 Rosetta Energy Units, consistent with eliminating the buried polar oxygen and improving the intra-residue contacts in that region. While most missense variants result in a destabilizing substitution, this type of hyper-stabilization could lead to a change-of-function effect including a) an increase in the steady-state protein level within the cell, or b) altered protein dynamics by favoring a specific conformation of this domain, leading to altered activity, binding, etc. (Figure 1B). Moreover, the location of the variant residue is unlikely to disrupt microtubule binding directly but may instead alter interactions between DCLK1 and other binding partners. The ability to retain binding to the microtubule but with altered interactions with other binding partners is a plausible mechanism for the change-of-function effect.

### *DCLK1* functional studies in *C. elegans*

*DCLK1* is highly conserved from humans to invertebrates. The *C. elegans* ortholog of *DCLK1* is *zyg-8*. The C-DCX domain of ZYG-8 shares ~60% similarity and 45% identity with DCLK1, and the affected proband residue, S228, is conserved as S382 in ZYG-8 (Figure 1C and Supplemental Figure S4A). This degree of conservation allowed us to use CRISPR-Cas9 genome editing to knock in the proband variant into the orthologous location in ZYG-8 to generate the *zyg-8*(S382L), hereafter referred to as *zyg-8(S382L)* (Figure 1D, and Supplemental Figure S4B). In addition, we generated a S382S control, hereafter referred to as *zyg-8(S382S)*, to account for the synonymous changes introduced to facilitate genotyping of CRISPR edits (Supplemental Figure S4B). Nonconditional, probable null alleles of *zyg-8* exist, but they have a tightly linked morphological marker, *unc-32(e189)* (Gönczy et al., 1999), which causes the animals to be uncoordinated and thereby precluding neuromuscular locomotion phenotyping. Therefore, we used CRISPR/Cas9 editing to generate a complete gene deletion from Start to Stop, hereafter referred to as *zyg-8(del)* or *zyg-8(−)*, for comparison (Supplemental Figure S4C).

#### The proband variant is damaging to *zyg-8* function:

Previous studies in *C. elegans* showed that ZYG-8 promotes microtubule growth and is required for proper mitotic spindle organization and positioning during early embryonic cell divisions. Consequently, loss-of-function mutations in *zyg-8* lead to defects in cell division and early embryonic lethality (Bellanger et al., 2007; Czajkowski et al., 2024; Gönczy et al., 2001; Srayko et al., 2005). To determine whether the proband variant is damaging *in vivo* we first assessed the functional impact of the proband variant on development. Consistent with *zyg-8* being a strict maternal effect embryonic lethal gene (Gönczy et al., 2001; Gönczy et al., 1999), *zyg-8(−/−)* mutants are viable in the first generation due to maternal rescue but are embryonic lethal in the second generation (Figure 1E–G). In comparison, animals homozygous for the proband variant, *zyg-8(S382L)*, are viable and fertile and are indistinguishable from the wild-type and *zyg-8(S382S)* control animals in terms of morphology, growth and a lack of embryonic lethality (Figure 1E–G). The *zyg-8(−/−)* embryonic lethality is, at least in part, from a failure in promoting microtubule growth in the anaphase mitotic spindle. The absence of embryonic lethality in the *zyg-8(S382L)* variant animals suggests that the variant does not cause a major defect in the microtubule growth function, although we cannot exclude the possibility that the variant is a hypomorph.

**Figure 2.**
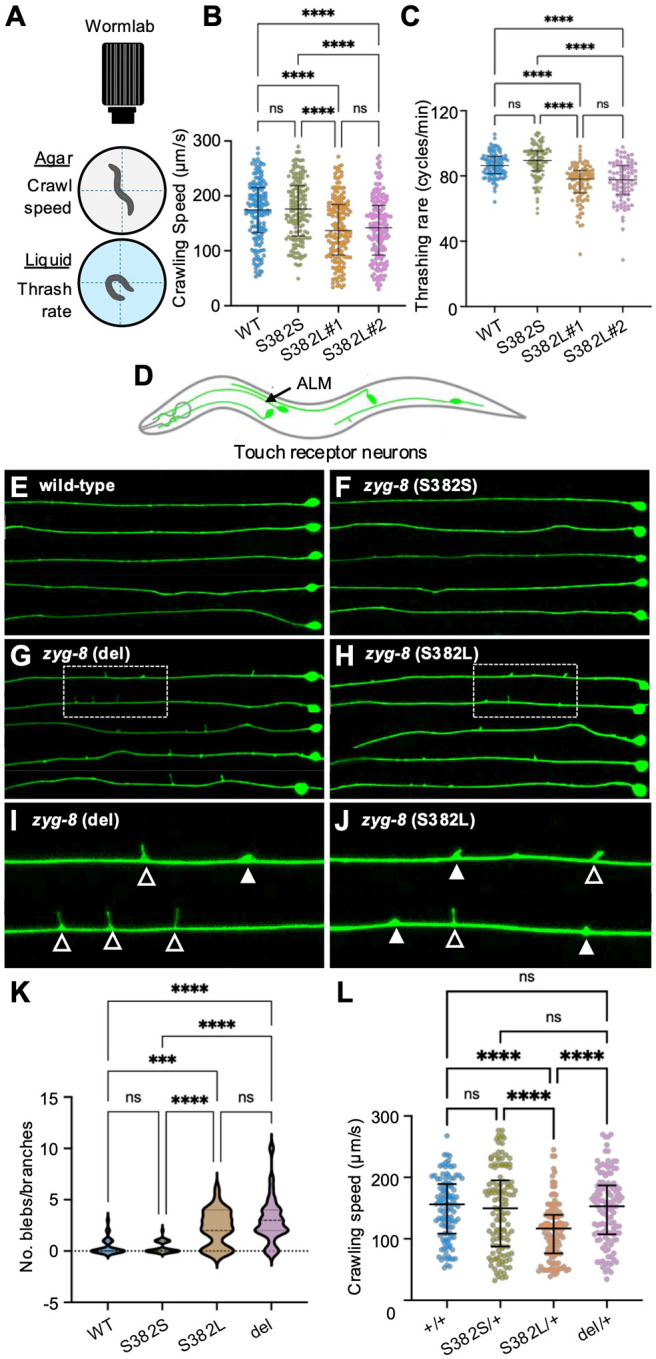
*DCLK1* p.(S228L) variant modeled in *C. elegans zyg-8(S382L)* is damaging. (A) Schematic of WormLab^®^ Imaging System used to quantify locomotion. *zyg-8(S382L)* is damaging to crawl speed and thrashing rate. Shown are median (B) crawl speed and (C) thrashing rates of wild-type (WT) (blue), homozygous *zyg-8(S382S)* control edit (green), and two independent lines, homozygous *zyg-8(S382L)#1* (orange) and homozygous *zyg-8(S382L)#2* variant animals (pink). Full genotypes, including allele designations are listed in Table S2. Three independent biological replicates were combined for each genotype. n= 30 per condition. Error bars indicate the median with interquartile range. Kruskal-Wallis test followed by Dunn’s multiple comparison test were used to determine statistical significance. ns, not significant. p-values <0.05 was considered statistically significant. **** indicates p-value < 0.0001. *zyg-8(S382L)* variant animals have abnormal touch receptor neuron (TRN) morphology. (D) Schematic of the position and projection of the six TRNs in *C. elegans* labeled using the mec-4::GFP transgene. Arrow points to ALM (L/R) neurons evaluated in this study. Fluorescent confocal images of ALM neurons of (E) wild-type, (F) *zyg-8(S382S)* control, (G) *zyg-8(del)* and, (H) *zyg-8(S382L)* variant animals. Higher magnification images of abnormal short branching and blebbing observed in (I) *zyg-8(del)* and, (J) *zyg-8(S382L)* variant animals. Empty triangles point to abnormal short branching and filled triangles point to neuronal blebs. (K) Quantification of the number of abnormal short branching and blebbing in each genotype. Three independent biological replicates were combined for each genotype. n=10 per condition. Error bars show mean with standard deviation. Differences between groups were determined using Kruskal-Wallis test followed by Dunn’s multiple comparison test. ns, not significant. p-values <0.05 were considered statistically significant. *** and **** indicate p-values < 0.001 and < 0.0001, respectively. (L) Quantification of heterozygous crawl speed. Three independent biological replicates were combined for each genotype. n=30 per condition. Error bars indicate the median with interquartile range. Kruskal-Wallis test followed by Dunn’s multiple comparison test were used to determine statistical significance. ns, not significant. p-values <0.05 was considered statistically significant. **** indicates p-value < 0.0001.

Next, we assessed the functional consequences of the proband variant on locomotion using the Wormlab^®^ Imaging System (MBF Bioscience), an automated video recording device for quantifying multiple movement parameters (Figure 2A) (Roussel et al., 2014). Crawling on solid agar surfaces and swimming (or thrashing) in liquid are two distinct modes of locomotion that are genetically regulated. Crawling and thrashing differ significantly in mechanics and frequency, and are sensitive and highly quantitative readouts for neuromuscular function (Fielder et al., 2025; Huang et al., 2022). The median crawling speeds of wild-type parents and *zyg-8(S382S)* control edit animals are 174 and 176 μm/s, respectively, consistent with previously published results (Figure 2B) (Fielder et al., 2025; Huang et al., 2022). In contrast, the two independent lines homozygous for the proband variant, *zyg-8(S382L)#1* and *zyg-8(S382L)#2*, crawled at significantly reduced speeds of 137 and 142 μm/s, respectively. Similarly, when placed in liquid, the two homozygous *zyg-8(S382L)* variant lines, thrashed (or swam) at significantly reduced rates compared to wild-type and *zyg-8(S382S)* controls (Figure 2C). Taken together, these results indicate that the proband variant, *zyg-8(S382L)*, is damaging to *zyg-8* function by disrupting locomotion.

#### The proband variant animals display neurological phenotypes:

Given that neurological phenotypes were observed in the proband, we assessed whether the homozygous *zyg-8(S382L)* animals exhibited any defects in neuronal morphology and growth. Similar to *DCLK1*, *zyg-8* is expressed extensively in neuronal structures in developing larvae and adults (Bellanger et al., 2007). In particular, *zyg-8* is expressed in numerous motoneurons as well as the six touch receptor neurons (TRNs) that sense mechanical stimuli (Chalfie, 2009). Loss-of-function mutations in *zyg-8* have been reported to cause defective TRN morphology characterized by the presence of varicosities which are abnormal blebs, bulges and short branches that are seen along the neurites. These varicosities were associated with a defect in touch sensitivity suggesting that the neurons were damaged or may have reduced function (Bellanger et al., 2007). To assess the functional consequences of the *zyg-8(S382L)* proband variant, we evaluated the morphology and growth of TRNs using the *mec-4p*::GFP transgene, which is exclusively expressed in the six TRNs. For this study, we focused on assessing the morphology of the ALM neurons (Figure 2D). High-resolution confocal imaging revealed significant number of blebs and abnormal short branches in the neurites of proband variant *zyg-8(S382L)* and *zyg-8(del)* animals that are absent in the wild-type and control *zyg-8(S382S)* animals (Figure 2E–K). These results indicate that the proband variant *zyg-8(S382L)* disrupts normal neuronal morphology.

#### The proband variant likely displays a change-of-function genetic mechanism:

Given the dominant inheritance pattern for the *DCLK1* variant, we examined whether heterozygous animals displayed dominant phenotypes. Heterozygous *zyg-8(S382L/+)* animals displayed a significantly reduced crawling speed (117 μm/s) compared to wild-type (156 μm/s) and *zyg-8(S382S/+)* (150 μm/s) controls (Figure 2L). In contrast, *zyg-8*(*del/+*) heterozygotes displayed a normal crawling speed (153 μm/s) similar to wild-type and control animals. The absence of a crawling speed phenotype in the *zyg-8*(*del/+*) heterozygotes indicates that *zyg-8* is not a haploinsufficient gene in *C. elegans*. Furthermore, the presence of a dominant phenotype in the *zyg-8(S382L/+)* heterozygous animals suggests that the genetic mechanism of the proband variant is a change-of-function and not a simple hypomorph.

Taken together, the functional studies in *C. elegans* provide strong evidence that the proband variant modeled in *zyg-8(S382L)* is damaging to gene function by disrupting locomotion and touch receptor neuron morphology.

#### Direct reprogramming efficiently generates patient-derived neurons (PDNs):

**Figure 3.**
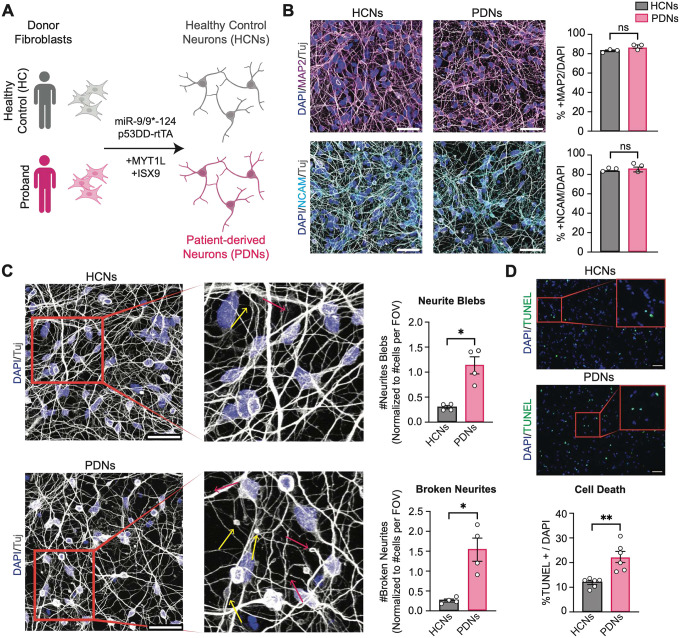
Cellular phenotypes in directly reprogrammed patient-derived neurons (PDNs) versus healthy control neurons (HCNs). (A) Schematic of the microRNA-mediated direct reprogramming system. (B) Representative confocal images and quantification of the mature neuronal markers MAP2 and NCAM in HCNs and PDNs (blue=DAPI, Tuj=grey, magenta=MAP2, cyan=NCAM). Quantification included three fields of view (FOV) (~30 neurons per field; ~90–100 neurons total) per condition. (C) Representative images (blue=DAPI, Tuj=grey) and quantification for neurite phenotypes in PDNs versus HCNs. The boxed region is shown at higher magnification, highlighting neurite blebs (yellow arrows) and broken or retracting neurites (pink arrows). Quantification using two FOV per coverslip across two coverslips (N=4) per condition. Data are mean ± SEM; points represent blebs or broken neurites normalized to the number of cells in the FOV, measured by DAPI. (D) Representative images and quantification of apoptosis by TUNEL. Insets show higher-magnification views of the boxed regions from the corresponding images. Three FOV per coverslip across two coverslips (N=6) per condition. Data are mean ± SEM; points represent percentage of TUNEL+/DAPI+ cells. Statistical significance determined using unpaired T-Test with Welch’s correction. ns = not significant, * = p<0.05, ** = p<0.01. Scale bars are 50 μm for all images.

Given that this is a single case, we sought additional evidence to link the abnormal neuronal morphology observed in the *C. elegans* model with the *DCLK1* cellular phenotype in the proband. To assess the functional impact of the proband’s variant within the human neuronal context, we generated patient-derived neurons (PDNs) via direct neuronal conversion of the proband fibroblasts, as well as age- and sex-matched healthy control neurons (HCNs) from control fibroblasts (GM05757; male 7 years; NIGMS Human Genetic Cell Repository), using the previously established microRNA-based cellular reprogramming protocol (Figure 3A) (Capano et al., 2022; Cates et al., 2025; Church et al., 2021). Briefly, fibroblasts were directly converted using miR-9/9* and miR124 (miR-9/9*−124) and MYT1L over 21 days (Cates et al., 2025) with ISX-9 treatment, which promotes cortical neuronal identity during the reprogramming via the activation of endogenous NEUROD-family transcription factors (Li et al., 2015). At post-induction day (PID) 23, immunostaining analysis revealed that >80% of DAPI-positive cells expressed neuronal markers MAP2 and NCAM in both HCNs and PDNs (Figure 3B), consistent with prior studies (Capano et al., 2022; Cates et al., 2021; Huh et al., 2016; Lee et al., 2024; Oh et al., 2022; Sun et al., 2024; Victor et al., 2014; Victor et al., 2018; Yoo et al., 2011). No significant difference in reprogramming efficiency, as marked by MAP2 or NCAM expression, was observed between HCNs and PDNs (Figure 3B), validating these directly reprogrammed neurons as a platform for assessing disease-associated phenotypes.

#### PDNs exhibit disease-associated neurite pathology and increased cell death:

Leveraging the phenotypic detection in the neurites of proband variant *zyg-8(S382L) C. elegans* (Figure 2), we first evaluated neuronal morphology in PDNs using high resolution confocal microscopy following immunostaining for the microtubule marker b3-tubulin (TUBB3, Tuj), which is highly expressed in neurons and frequently used as a marker for neurite morphology. At PID 23, PDNs displayed marked disruption of the neurite network compared to HCNs with prominent neurite blebbing characterized by beading and swelling (Figure 3C). Quantitative analysis of independently reprogrammed neuronal populations from PDNs and HCNs revealed a significant increase in neurite blebs and broken or retracting neurites in PDNs relative to HCNs (Figure 3C). Both dystrophic neurites and impaired neurite integrity have been shown to reflect diminished neuronal resiliency to stress (Dickson et al., 1999; Onorato et al., 1989; Reza-Zaldivar et al., 2020; Sadleir et al., 2016). Compared to HCNs, PDNs demonstrated an increased number of TUNEL positive cells, consistent with increased cell death (Figure 3D). Together, these findings demonstrate that PDNs exhibit impaired neurite integrity, recapitulating the neurite blebbing phenotypes observed in the *C. elegans* model (Figure 2E–J), as well as increased cell death.

#### Transcriptomic profiling reveals disease-relevant pathways in PDNs:

**Figure 4.**
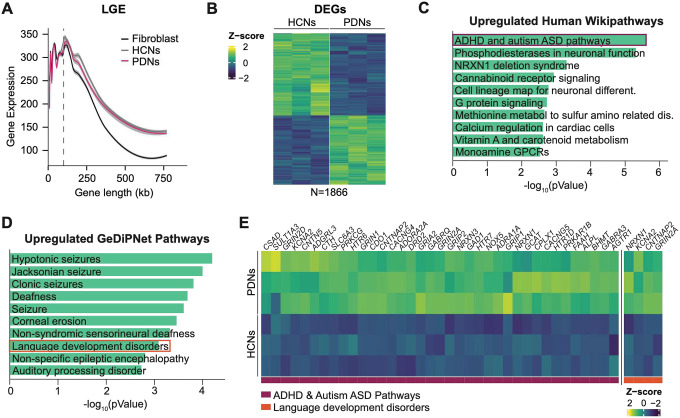
Transcriptomic profiling of patient-derived neurons (PDNs) reveals disease-associated pathways. (A) LONGO analysis demonstrating increased long gene expression (LGE) after direct conversion in both healthy control neurons (HCNs) and PDNs compared to fibroblasts (B) Heatmap showing global gene expression differences between HCNs and PDNs. (C) Top enriched Human Wikipathways from upregulated differentially expressed genes (DEGs) in PDNs compared to HCNs. (D) Top enriched GeDiPNet pathways from upregulated DEGs in PDNs compared to HCNs. (E) Heatmap for DEGs within highlighted disease-relevant pathways. ADHD = attention deficit/hyperactivity disorder. ASD = autism spectrum disorder.

To further define the molecular impact of the proband *DCLK1* variant, we performed comparative transcriptome analysis using bulk RNA-sequencing (RNA-seq) between HCNs and PDNs. To first assess the transcriptome signature of mature neurons, we analyzed long gene expression (LGE; >100kb), a transcriptomic hallmark unique to mature neurons(Gabel et al., 2015; King et al., 2013; Lu et al., 2021). Using LONGO analysis (McCoy et al., 2018), we found that both HCNs and PDNs exhibited increased LGE in comparison to fibroblasts, with no difference observed between groups (Figure 4A), supporting comparable neuronal conversion efficiency and neuronal maturity between HCNs and PDNs. Notably, the similar LGE detected between HCNs and PDNs also argues against a role for the proband *SFPQ* variant, as loss-of-function in SFPQ is associated with decreased LGE (Takeuchi et al., 2018).

Differential expression analysis identified 1866 differentially expressed genes (DEGs; |log_2_FC| > 0.5, FDR < 0.05) between HCNs and PDNs (Figure 4B). Pathway analysis of genes upregulated in PDNs compared to HCNs identified enrichment of neurodevelopmental disorder-associated pathways, including ADHD and autism (ASD) pathways, seizure disorder pathways, and language development disorder pathways (Figure 4C, D). DEGs within ASD pathways and language development disorders include *KCNA2*, *GABRA3, GABRQ, GRIN2A, HTR7, NRXN3, NRXN1, CNTNAP2* and *GRIP2* (Figure 4E), all of which are previously implicated in neurodevelopmental disorders (Amador et al., 2020; Griswold et al., 2012; Johannesen et al., 2026; Mejias et al., 2011; Mejias et al., 2019; Piton et al., 2013; Strehlow et al., 2019; Syrbe et al., 2015; Xie et al., 2024). Importantly, differential expression of these genes may represent the neuronal state relevant to the proband’s clinical features of autism spectrum disorder and developmental regression.

Interestingly, relevant to the dystrophic neurite phenotypes in PDNs, we also detected significant upregulation of axon guidance genes, *RGMA* (log_2_FC = 1.1, FDR = 1.07 × 10^−29^) *SEMA5B* (log_2_FC = 1.67, FDR = 0.001) in PDNs compared to HCNs. RGMa (Repulsive Guidance Molecule A) induces growth cone collapse and inhibits neurite outgrowth (Endo & Yamashita, 2009; Lah & Key, 2012; Matsunaga et al., 2006) and has recently been implicated in the pathogenesis of neurodegenerative diseases, including Parkinson’s disease (PD) and amyotrophic lateral sclerosis (ALS) (Korecka et al., 2017; Oda et al., 2021; Shimizu et al., 2023). SEMA5B, a semaphorin family member, similarly functions as a repulsive axon guidance cue during neurodevelopment and can disrupt synaptic connectivity when overexpressed (Jung et al., 2019; Lett et al., 2009; O’Connor et al., 2009).

Conversely, genes downregulated in PDNs were enriched for GO BP terms related to extracellular matrix organization, cell migration, and inflammatory signaling (Figure S5A). Consistent with this, Wikipathways and MSigDB Hallmark analyses demonstrated reduced inflammatory and cytokine signaling pathways, including epithelial mesenchymal transition, TNF-α signaling via NF-kB, interferon gamma response, and interferon alpha response pathways (Figure S5B,C). Overall, as DCLK1 has been linked to these pathways in non-neuronal contexts (Chandrakesan et al., 2016; Chandrakesan et al., 2014; Luo et al., 2023), these data suggest that PDNs capture transcriptional programs relevant to DCLK1 function in neurons.

#### Wild-type *DCLK1 (WT-DCLK1)* overexpression partially rescues cellular phenotypes in PDNs:

**Figure 5.**
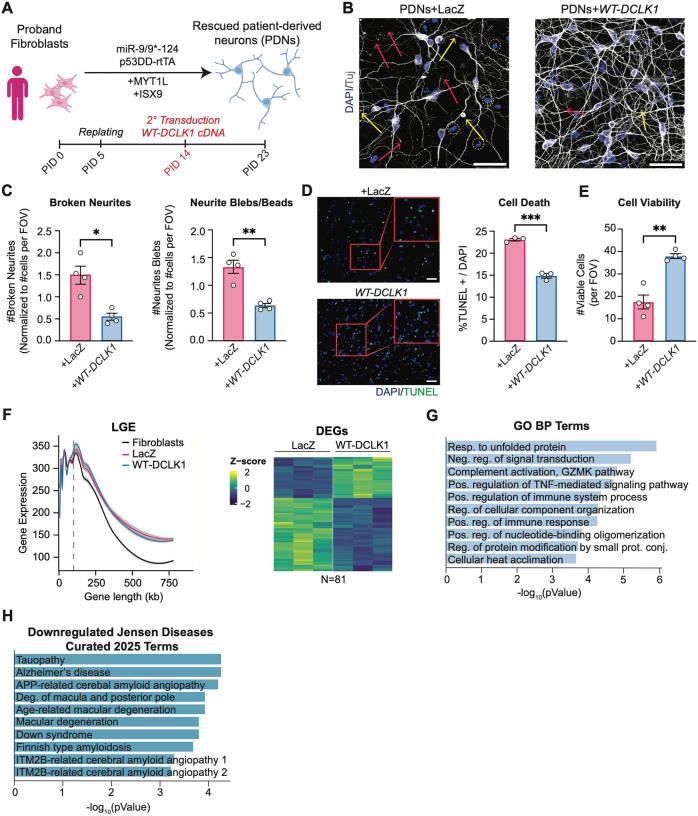
*WT-DCLK1* overexpression partially rescues cellular and transcriptional phenotypes in patient-derived neurons (PDNs). (A) Schematic of direct reprogramming and *WT-DCLK1* overexpression. (B) Representative images (blue=DAPI; Tuj=grey) of PDNs expressing LacZ or *WT-DCLK1*. *WT-DCLK1* reduces neurite blebbing (yellow arrows), fragmentation (pink arrows), and cell death (yellow circles). (C) Quantification of neurite defects using two fields of view (FOV) per coverslip across two coverslips (total N=4) per condition. Data are mean ± standard error mean (SEM); points represent blebs or broken neurites normalized to the number of cells in the FOV, measured by DAPI. (D) TUNEL staining and quantification of apoptosis with three FOV per condition (N=3). Insets show higher-magnification views of the boxed regions from the corresponding images. Data are mean ± SEM; points represent the percentage of TUNEL+/DAPI+. (E) Quantification of cell viability using three FOV per condition (N=3). Viable cells were defined as non-fragmented DAPI+ cells with extending Tuj+ neurites. Data are mean ± SEM; points represent the number of viable cells per FOV. (F) LONGO analysis showing comparable long-gene expression (left) and heatmap of global transcriptional changes (right) between LacZ and *WT-DCLK1* conditions. (G) Top enriched GO BP terms from all differentially expressed genes (DEGs) between LacZ and *WT-DCLK1* PDNs. (H) Disease-related pathways downregulated in *WT-DCLK1* compared to LacZ PDNs. Statistics: unpaired t-test with Welch’s correction; *p<0.05, **p<0.01, ***p<0.001. Scale bars are 50 μm for all images.

To further test the functional contribution of the proband *DCLK1* variant, we performed rescue experiments by overexpressing *WT-DCLK1* cDNA in PDNs during reprogramming. Secondary transduction for cDNA overexpression for *WT-DCLK1* and LacZ (control) was performed around PID10–14 (Figure 5A), a reprogramming timeframe that has previously shown to permit robust transgene expression without disrupting neuronal fate acquisition (Cates et al., 2025; Oh et al., 2022; Sun et al., 2024). Neurite morphology and cell death phenotypes were then compared between the LacZ and *WT-DCLK1* PDNs. We found that *WT-DCLK1* overexpression markedly improved neurite integrity, with a visible reduction in dystrophic neurites (Figure 5B). Quantitative analysis showed significant decreases in the number of cells with neurite blebbing and broken or retracting neurites, as well as decreased TUNEL positive cells and an increased number of viable cells compared to LacZ controls (Figure 5C–E). These findings strongly suggest that the proband’s *DCLK1* variant impairs neurite homeostasis and neuronal resiliency, and that *WT-DCLK1* expression is sufficient to at least partially rescue these phenotypes.

#### *WT-DCLK1* overexpression partially restores gene expression changes in PDNs:

To determine the transcriptional impact of *WT-DCLK1* rescue, we performed RNA-seq on PDNs expressing *WT-DCLK1* or LacZ. *WT-DCLK1* overexpression induced a shift in global gene expression, while LGE remained unchanged between conditions (Figure 5F; left). Differential expression analysis identified 81 DEGs (|log_2_FC| > 0.5, FDR < 0.05) between LacZ and *WT-DCLK1* PDNs (Figure 5F; right). Functional enrichment analysis of all DEGs revealed pathways related to cell stress responses, including response to unfolded protein, complement activation, positive regulation of TNF-α signaling, and cellular heat acclimation (Figure 5G). Interestingly, analysis of DEGs downregulated in *WT-DCLK1* PDNs revealed pathways implicated in neurodegenerative disorders, including tauopathy, Alzheimer’s disease, age-related macular degeneration, and Down syndrome (Figure 5H). These findings are consistent with the observed rescue of neurite integrity and cell survival, supporting the role of *WT-DCLK1* in modulating neuronal stress and resilience pathways.

#### *WT-DCLK1* restores a subset of disease-relevant genes towards HCNs:

**Figure 6.**
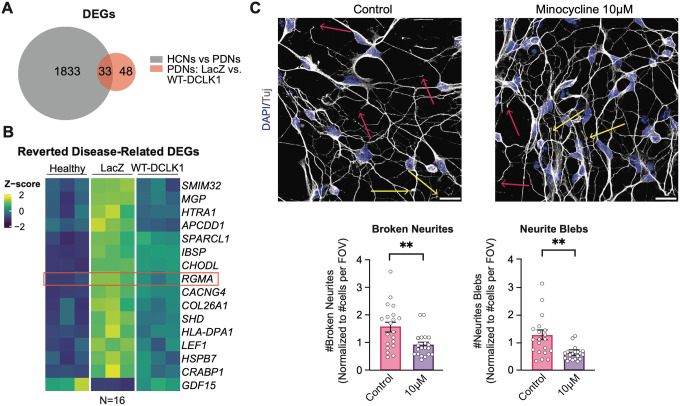
Partial rescue of abnormal neurite phenotypes in patient-derived neurons (PDNs) with minocycline treatment. (A) Overlap of differentially expressed genes (DEGs) across comparisons and identification of 33 common DEGs. (B) Heatmap illustrating partial restoration of gene expression toward healthy control neuron (HCN) expression levels with *WT-DCLK1* overexpression, including *RGMA* (Repulsive Guidance Molecule A). (C) Representative images (blue=DAPI, Tuj=grey) and quantification of neurite blebs (yellow arrows) and broken or retracting neurites (pink arrows) in control versus minocycline treated PDNs. Quantification using ten fields of view (FOV) per coverslip across two coverslips (N=20) per condition. Data are mean ± SEM; points represent blebs or broken neurites normalized to the number of cells in the FOV, measured by DAPI. Scale bars are 50 μm for all images. Statistics: unpaired t-test with Welch’s correction; **p<0.01.

To identify genes associated with phenotypic rescue, we intersected DEGs from HCNs versus PDNs and LacZ versus *WT-DCLK1* PDNs. This analysis identified 33 shared DEGs (|log_2_FC| > 0.5, FDR < 0.05), of which 16 shifted towards HCN expression levels following *WT-DCLK1* overexpression (Figure 6A,B). Notably, *RGMA* was among the reverted genes and was significantly downregulated in the *WT-DCLK1* rescue condition (log_2_FC = −0.55, FDR = 2.62 × 10^−7^). This is intriguing as inhibition of RGMA has been shown to be neuroprotective in animal models of ALS and PD (Oda et al., 2021; Shimizu et al., 2023). Conversely, *GDF15*, a TGF-b family member with established neuroprotective roles (Machado et al., 2016; Strelau et al., 2000; Subramaniam et al., 2003), was upregulated following *WT-DCLK1* overexpression (log_2_FC = 0.62, FDR = 1.85 × 10^−7^), reversing its downregulation in PDNs. Together, these results indicate that *WT-DCLK1* partially restores disease-associated transcriptional programs in the patient neurons, including genes implicated in neurite regulation and neuronal survival, providing a molecular basis for the observed phenotypic rescue.

#### Treatment of PDNs with minocycline modifies neurite phenotypes:

We next tested whether neurite phenotypes could be modified through pharmacologic treatment. Previous research has demonstrated that targeted inhibition of RGMA can promote axonal growth post spinal cord injury (Nakagawa et al., 2019), improve neurologic symptoms in rodent models of multiple sclerosis (Demicheva et al., 2015), and provide neuroprotective effects in animal models of ALS and PD (Oda et al., 2021; Shimizu et al., 2023). As *RGMA* was identified as a key gene that was downregulated and reverted towards HCN levels in PDNs following *WT-DCLK1* overexpression (Figure 6B), we tested whether chemical inhibition of RGMA with minocycline would affect neurite morphology. Previous studies have shown that minocycline can suppress expression of RGMA in microglia (Kitayama et al., 2011) and that treatment of rats with minocycline decreases RGMA expression, decreases axonal damage, and improves neurologic recovery post ischemic stroke (Tao et al., 2013). Quantitative analysis of independently reprogrammed neuronal populations from PDNs revealed a significant decrease in neurite blebs and broken or retracting neurites with minocycline treatment (Figure 6C). These data suggests that the abnormal neurite phenotypes observed in PDNs may be modifiable pharmacologically, in addition to rescue with *WT-DCLK1* overexpression.

## DISCUSSION

In this study, we leverage the multidisciplinary framework of the Undiagnosed Diseases Network (UDN), as well as neurons directly reprogrammed from patient-specific fibroblasts, to establish *DCLK1* as a candidate gene associated with a previously unrecognized progressive neurodevelopmental disorder. Our findings support a model in which the *DCLK1* p.(S228L) variant contributes to the neurodevelopmental disorder by perturbing neuronal homeostasis through a dominant change-of-function mechanism. *C. elegans* heterozygous for the proband variant displayed significant locomotor impairment, whereas heterozygous deletion animals were phenotypically normal arguing against a simple haploinsufficiency model. Structural modeling further supports a change-of-function mechanism by predicting increased stability of the second DCX domain containing p.S228L, rather than decreased structural stability that is typical of most missense changes leading to reduction of protein function. Given that the affected residue is positioned outside the predicted microtubule-binding interface, the variant may instead alter interactions with regulatory or scaffolding proteins necessary for neuronal maintenance and neurite integrity. Future biochemical studies examining DCLK1 binding partners and microtubule dynamics will be important for clarifying the precise molecular consequences of this variant.

The concordance between the *C. elegans* model and patient-derived neuronal studies provides strong support that the *DCLK1* p.(S228L) variant is damaging and likely contributes to the proband phenotype. The neurite blebbing and abnormal branching observed in *C. elegans zyg-8(S382L)* animals were similarly observed in patient-derived neurons (PDNs), which also exhibited dystrophic neurites, and increased cell death. These features are well-recognized indicators of neuronal injury and impaired resiliency and have been implicated across a range of neurodevelopmental and neurodegenerative disorders, providing an explanation for the neurodevelopmental regression observed in the patient (Dickson et al., 1999; Onorato et al., 1989; Sadleir et al., 2016; Sun et al., 2024). These findings also highlight the importance of employing age-relevant, cell culture neuronal modeling systems for functional evaluation of candidate disease-associated genes in regressive and/or degenerative progression of neurologic disorders, as demonstrated for disease stage-dependent modeling of Huntington’s disease and late-onset Alzheimer’s disease (Lee et al., 2024; Oh et al., 2022; Sun et al., 2024).

Transcriptomic profiling of PDNs further supports the biological relevance of the observed phenotypes. Particularly notable was the enrichment of neurodevelopmental disorder-associated pathways including ADHD and autism (ASD) pathways relevant to the proband and the upregulation of repulsive axonal guidance genes, *RGMA* and *SEMA5B*, recently implicated in the pathogenesis of neurodegenerative disease. Dysregulation of these genes/pathways may contribute mechanistically to the neurite degeneration phenotype observed in PDNs. Importantly, overexpression of wild-type *DCLK1* partially reverted the dystrophic neurite phenotype and partially restored *RGMA* expression while increasing expression of the neuroprotective factor *GDF15*, suggesting that *DCLK1* may influence neuronal resilience through modulation of stress-response and axon-guidance signaling pathways. We also found that treatment of PDNs with minocycline, which has previously been utilized to inhibit RGMA, improves dystrophic neurite phenotypes. This suggests that the neurite defects may be chemically/pharmacologically modifiable and strengthens the potential translational applications of the multi-modal modeling approach utilized in our study.

Our study also highlights the diagnostic challenges posed by unresolved cases of neurodevelopmental disorders in the setting of multiple candidate variants. Despite strong *in silico* predictions for both *DCLK1* and *SFPQ*, cross-species functional analyses enabled prioritization of *DCLK1* as the more likely disease-associated gene. While a contributory role for *SFPQ* p.(Pro623Arg) cannot be excluded, several lines of evidence argue against a primary pathogenic role, including the absence of detectable variant-specific phenotypes in *Drosophila* and the preservation of long gene expression (LGE) signatures in patient-derived neurons, one of the known functions of SFPQ.

More broadly, this work demonstrates the value of integrating model organism genetics, structural biology, and patient-derived cellular systems to resolve variant interpretation in rare disease research. The *C. elegans* findings indicating that *DCLK1* p.(S228L) is likely damaging, motivated the more involved studies with patient-specific cellular modeling using directly reprogrammed neurons. Importantly, miRNA-mediated direct reprogramming propagates age signatures from the starting fibroblasts (Huh et al., 2016; Lu et al., 2021), making this approach particularly useful for identifying age- and disease stage-dependent neuronal pathologies in a delayed onset neurodevelopmental regressive disorder. The cellular phenotypes observed in directly reprogramed neurons have recapitulated age-relevant and disease stage-dependent neuropathology for multiple neurodegenerative diseases (Capano et al., 2022; Lee et al., 2024; Oh et al., 2022; Sun et al., 2024; Victor et al., 2018), and thus the dystrophic neurite phenotype we observed in patient neurons is likely a feature of age-relevant patient’s cellular pathology. Such multidisciplinary strategies will likely become increasingly important as genomic sequencing continues to identify ultra-rare variants without established disease associations.

Several limitations of the current study should also be acknowledged. Most notably, this report describes a single individual, and identification of additional individuals with *DCLK1* variants will be necessary to establish a definitive gene–disease relationship and better define the phenotypic spectrum. In addition, while the rescue experiments support a role for *DCLK1*, the partial nature of rescue suggests additional complexity, at the experimental and/or the genetic level. Although minocycline treatment partially rescued neurite defects in PDNs, additional studies are needed to define the molecular mechanisms underlying these effects. Minocycline has previously been reported to inhibit RGMA signaling and promote axonal integrity, but it also exerts a range of neuroprotective actions, including anti-inflammatory, antioxidant, and anti-apoptotic effects. Consequently, the specific pathways responsible for the observed improvement in PDN neurite morphology remain unclear. Nevertheless, these findings are significant because they demonstrate that disease-associated neuronal phenotypes are not only responsive to genetic rescue through wild-type *DCLK1* expression, but may also be amenable to pharmacologic intervention. Finally, the precise biochemical consequences of the S228L substitution remain unresolved and warrant future investigation.

In summary, we provide convergent genetic and functional evidence implicating *DCLK1* p.(S228L) in a previously unrecognized neurodevelopmental disorder. Through integration of computational protein modeling, *in vivo* functional studies in model organisms, and patient-derived neuronal analyses, we demonstrate that the *DCLK1* p.(S228L) variant disrupts neuronal morphology/function and transcriptional programs through a likely dominant change-of-function mechanism. These findings broaden the spectrum of doublecortin gene family-related disorders and underscore the importance of integrative functional experimental approaches for establishing causality in rare neurodevelopmental disease.

## MATERIALS & METHODS

### Informed consent

Proband and parents were enrolled into the Undiagnosed Diseases Network (UDN) in an effort to identify an underlying genetic cause for the clinical phenotype. The protocol is overseen by the National Institutes of Health IRB (15HG0130). Informed consent was obtained before any study procedures.

### Sequencing and variant analysis

Exome sequencing (ES) was completed through a clinical testing laboratory, and the sequencing data were obtained by the UDN team for additional analyses. Codified Genomics software was used for variant prioritization (www.codifiedgenomics.com). *De novo*, X-linked, and biallelic variants with allele frequencies of less than 1% in gnomAD were prioritized. The *de novo* variants of interest identified from ES reanalysis, which were not on the clinical report, were subsequently confirmed via Sanger sequencing.

### *Drosophila* modeling

#### Generation of *SFPQ* transgenic fly lines

The human SFPQ transgenic lines were generated as described previously (Harnish et al., 2019). The full-length reference SFPQ cDNA sequence (RefSeq: NM_005066.3) was codon-optimized for *Drosophila* expression and inserted into pUAST.attB expression vector through gene synthesis (TWIST Biosciences, CA, USA). Similarly, the variants reported in this paper were synthesized using the same platform. The entire plasmid sequence was validated by whole-plasmid sequencing (Plasmidsaurus, CA, USA). These plasmids were microinjected into the VK00037 attP docking site through phiC31-mediated transgenesis (Venken et al., 2006), and the transgenic lines were balanced using a SM6a balancer chromosome and two independent transgenic lines we established for functional studies for each transgene.

#### Fly stocks and maintenance

All the flies were grown on a standard medium consisting of water, yeast, soy flour, cornmeal, agar, corn syrup, and propionic acid. Fly stocks were maintained in 18 °C temperature-controlled rooms or 25 °C incubators with 50% humidity. Crossed flies were raised in a 25 °C incubator, otherwise specified. All the flies were given 12-hour light/12-hour dark cycles.

#### Western blotting for SFPQ

The *UAS-SFPQ* reference and variant transgenic flies were crossed with *heat shock-Gal4* (Mok et al., 2025) driver line. Flies were raised at room temperature before eclosion. After eclosion, adult flies were given a heat shock at 37 °C for 30 minutes to induce transgene expression. Then, flies were placed in a 29 °C incubator for 24 hours. Ten female whole flies were homogenized with lysis buffer [1% TritonX-100, 25 mM Tris-HCl (pH=7.5), 100 mM NaCl, 1 mM EDTA with a protease inhibitor cocktail (K1007, APExBIO, TX, USA)]. Samples were centrifuged for 20 minutes at 14,000rpm, 4 °C, and the supernatant was collected for SDS-PAGE. They were mixed 1:1 with Laemmli sample buffer (Bio-Rad, CA, USA) with 10% 2-merchaptoethanol and boiled at 95 °C for 10 minutes. Samples were separated using a premade 4–20% gradient gel (Bio-Rad) and transferred onto the polyvinylidene fluoride (PVDF) membranes by wet transfer using eBlot L1 (Genscript, NJ, USA). The membranes were incubated overnight at 4 °C with primary antibodies, anti-HA (11867423001, Millipore Sigma, MA, USA) 100mg/ml, or/and anti-b-actin (C4/MAB1501. Millipore Sigma,) 1:1000. The next day, the membranes were incubated with secondary antibodies, Goat anti-Mouse IgG (H+L) Cross-Adsorbed Secondary Antibody HRP (G21040, Invitrogen, CA, USA) 1:20,000 or Peroxidase AffiniPure Goat anti-rat IgG+IgM (H+L) (112–035-044, Jackson Immunoresearch, PA, USA) 1:20,000, for two hours at room temperature. The chemiluminescent signal was detected using Immobilon Crescendo Western HRP substrate (WBLUR0100, Millipore Sigma) by ChemiDocTM MP (Bio-Rad). Quantification was performed using ImageJ, and statistical analysis (one-way analysis of variance (ANOVA) followed by Dunnett’s multiple comparisons test) was conducted using Prism 11 software.

#### Protein structural modeling

The impact of the S228L variant was assessed using the VUStruct webserver (Moth et al., 2025). None of the 11 experimental structures available for Serine/threonine-protein kinase DCLK1 (UniProt ID O15075) include the second (C-DCX) doublecortin domain (residues 186–269) where the S228L variant is located. Therefore, the domain was taken from the full-length AlphaFold (Jumper et al., 2021; Varadi et al., 2024) model AF-O15075-F1 and the MODBASE (Pieper et al., 2014) model ENSP00000353846.3. The model of the domain was considered in isolation, as the inter-domain information involving the C-DCX domain of DCLK1 is sparse, as evidenced by poor PAE scores in the AlphaFold model. The average pLDDT score of the AlphaFold model is 71, indicating moderate to high confidence in the overall backbone structure, but lower confidence in the detailed side-chain conformations, preventing us from making any claims about specific side-chain interactions in the models (e.g. gain or loss of a specific hydrogen bond).

#### *C. elegans* strain and culture conditions

*C. elegans* strains were cultured and maintained on an OP50 *E. coli* lawn on nematode growth medium (NGM) at 20°C (Brenner, 1974). A full list of strains used in this study is in Supplemental Table S2. Some strains were provided by the CGC, which is funded by NIH Office of Research Infrastructure Programs (P40 OD010440).

#### *C. elegans* CRISPR/Cas9 gene editing

CRISPR-Cas9 genome editing in *C. elegans* was performed as previously described (Fielder et al., 2025; Huang et al., 2022). In brief a mixture of a guide RNA (gRNA), DNA repair template with the corresponding nucleotide changes, and the Cas9 protein was microinjected in gravid adults from wild type VC2010. The pRF4 was included to use as a co-injection marker to identify the worms with successful Cas9 editing. The repair templates were designed to have about 33-nt homology arms on each side with synonymous changes to prevent gRNA rebinding and create a new restriction enzyme cleavage site to assist in genotyping. The edited lines referred as control edits were generated by the repair templates without proband variant to verify that the silent mutations did not contribute to the observed phenotype (referred as S382S). Edited progeny from different injected mothers were considered as independent lines. The *zyg-8* gene was Sanger sequenced and confirmed to have all designed variants but no extraneous changes in the gene. All strains were backcrossed with VC2010 at least twice to remove random mutations introduced by CRISPR editing. Three independent variant lines and two independent control lines were obtained and used to study potential phenotypes. A null allele was generated by deleting the entire *zyg-8* gene from Start to Stop. The gRNAs, repair templates and PCR primers used for genotyping are listed in Supplementary Table S3.

#### *C. elegans* phenotypic assays

##### Growth rate:

For each genotype, 20 L4 stage F1 homozygotes animals were placed onto a 60mm NGM plate and incubated at 20 °C overnight then were transferred to a new plate to lay eggs for 2 hours. 100 F2 embryos from each genotype were transferred to a new plate. Development of F2 homozygotes were scored after 72 hours. The number of all stage animals including dead embryos on each plate was recorded and totaled. The representative images from each genotype were taken by upright Zeiss compound microscope. Experiments were repeated a minimum of three times.

##### Locomotion:

Crawling speed and thrashing (swimming) were measured using the WormLab^®^ Imaging System as described (Fielder et al., 2025). The embryos were collected as eggs laid for 4 hours at 20°C, then shifted to 25°C and developed to L4 stages. The locomotion phenotypes were scored by Wormlab system (MBF bioscience) 24 hours after the late L4 stage grown at 25°C. For experiments using homozygotes, animals were picked from self-progeny plates. For Wormlab experiments using heterozygotes, the animals were obtained by *fem-1* RNAi treated females crossed with wild-type males. The heterozygous progenies were used. Briefly, 35 late L4s per strain were picked 24 hours before the assay. On the day of assay 10 animals were transferred to each assay plate, allowing them to crawl for 20 minutes. The videos were recorded 2 minutes for crawling and 1 minute for thrashing in 1XPBS. Three plates per strain were recorded per trial and three independent trials were performed to each experiment in which up to ~90 animals were assessed per strain. The videos were processed and analyzed using Wormlab software.

##### Neurite morphology:

A transgenic line expressing mec-4::GFP in touch neuron receptors was crossed into control, variant and deletion lines. For imaging, worms with the mec-4::GFP markers were immobilized with 5ul of 10mm levamisole in a 35 mm cover glass bottom dish (MatTek) and covered with a 12-mm circular coverslip and then a 25-mm square coverslip. Approximately 20 24-hour adult animals were transferred. Confocal images were taken with a Lecia SP8X tandem scanning confocal microscope with a white light laser using either a 40x 1.3 NA oil PlanApo objective over ≥20 z-planes and a pinhole size of 1.00 (Leica Microsystems). Images were displayed as maximum intensity projections. All images were rendered and analyzed using LASX (Leica Microsystems) and ImageJ software. The varicosities on each ALM were manually counted from four genotypes.

#### Fibroblasts and cell culture

Patient fibroblast samples were obtained through the Undiagnosed Diseases Network (UDN). The healthy control fibroblast line (GM05757; male 7 years; NIGMS Human Genetic Cell Repository) was obtained from the Coriell Institute. All fibroblasts were maintained in Dulbecco’s Modified Eagle Medium (DMEM, Invitrogen) containing 10% FBS supplemented with 0.01% β-mercaptoethanol, 1% non-essential amino acids, 1% sodium pyruvate, 1% 1M HEPES, 1% Glutamax^™^, and 1% penicillin/streptomycin solution (all from Gibco).

#### Lentivirus production

Lentiviruses were generated as previously described (Church et al., 2021). Briefly, viral supernatant was collected following transfection, centrifuged at 1,200g for 5 min at 4°C, and filtered through a 0.45 μm filter. The clarified supernatant was incubated overnight at 4°C with Lenti-X^™^ Concentrator (Takara Bio Inc.), followed by centrifugation at 1,200g for 45 min at 4°C. For doxycycline-inducible miR-9/9*−124, dominant-negative p53 (p53DD) with reverse tetracycline-controlled transactivator (rtTA), and MYT1L constructs, viral pellets were resuspended in DMEM supplemented with 10% FBS to generate single-concentrated lentivirus. For WT-DCLK1 overexpression and LacZ control vectors, viral pellets were resuspended in PBS at one-tenth of the original volume, overlaid onto a sucrose cushion (20% sucrose, 100 mM NaCl, 20 mM HEPES, 1 mM EDTA), and ultracentrifuged at 70,000 × g for 2 hours at 4°C. Pellets were then resuspended in one-hundredth of the original volume in 10% sucrose, 25 mM HEPES in PBS to generate double-concentrated lentivirus for secondary transduction. All viral preparations were stored at −80 °C and used within 1 year of production.

#### Direct neuronal reprogramming

Directly reprogrammed neurons were generated from human fibroblasts as previously described with minor modifications (Capano et al., 2022; Cates et al., 2025; Church et al., 2021). Fibroblasts were transduced with a single-concentrated lentiviral cocktail containing doxycycline-inducible miR-9/9*−124, p53DD-rtTA, and MYT1L. On post-induction day (PID) 1, media was replaced with fresh DMEM supplemented with 10% FBS and 1 μg/mL doxycycline (2 mL per well). On PID3, media was refreshed and supplemented with doxycycline and 3 μg/mL puromycin for selection. Cells were replated onto acid-treated, poly-ornithine/laminin/fibronectin-coated glass coverslips on PID5. On PID6, cultures were switched to Neurobasal^™^-A medium supplemented with GlutaMAX^™^ (500X) and B-27^™^ Plus (50X) (all from Gibco), containing doxycycline, 200 μM dibutyryl cyclic AMP, 1 mM valproic acid, 10 ng/mL BDNF, 10 ng/mL NT-3, 1 μM retinoic acid, RevitaCell^™^ Supplement (100X), and 3 μg/mL puromycin. Cultures were half-fed every 4 days, with doxycycline replenished on alternating 2-day intervals. To enhance cortical neuronal identity, cells were treated with the NeuroD1 activator ISX9 (10 μM) on PID3, PID6, PID10, and PID14. For secondary transduction, cells were transduced with double-concentrated lentivirus (250X) between PID10 and PID14. Supplementation with RevitaCell^™^, ISX9, and puromycin was discontinued after PID14. For minocycline experiments, cells were treated with minocycline (10μM) every two days from PID10 to completion of reprogramming. Previous studies have demonstrated minocycline 10μM to be effective and well tolerated, with higher doses demonstrating significant toxicity (Matsukawa et al., 2009; Tao et al., 2017).

#### Immunostaining and antibodies

Cells were fixed with 4% paraformaldehyde at room temperature for 20 min. Following permeabilization with PBS containing 0.2% Triton X-100 for 10 min, cells were blocked with blocking buffer (1% goat serum, 5% BSA in PBS) for 1 hour at room temperature. Primary antibodies were incubated overnight in blocking buffer at 4°C. Cells were then washed thrice with PBS and incubated with secondary antibodies in blocking buffer for 1 hour at room temperature. Cells were washed with PBS twice and then incubated with DAPI (Sigma) for 10 min before mounting to coverslips with a drop of anti-fade prolong gold reagent (Life technology) for imaging. Confocal images were obtained using a Leica SP5X white-light laser confocal system and processed using ImageJ. Primary antibodies used included mouse anti-NCAM (Santa Cruz, SC-106 1:50), rab anti-MAP2 (Cell Signaling, #4542 1:250), chicken anti-TUBB3/Tuj (Novus, NG100–1612 1:1000).

#### TUNEL cell death assay

Apoptotic cells were detected using the Promega DeadEnd^™^ Fluorometric TUNEL System (Promega Product # G3250) based on the manufacturer’s protocol. Following fixation, cells with permeabilized 0.2% Triton X-100 for 5 min and then washed with PBS twice. Coverslips were then covered with 100μl of Equilibration Buffer and rocked for 8 min at room temperature. After complete removal of the buffer, 51μl of the terminal deoxynucleotidyl transferase (TdT) reaction mix was added on top of the coverslip. Coverslips were then incubated at 37°C for 30 minutes while wrapped in foil to protect from light. The reaction was stopped by rocking the coverslips with 2X SSC buffer for 15 min. Following two PBS washes, cells were counterstained with DAPI for 5 mins. Images were taken using a Leica Mateo FL Digital Fluorescence Microscope and analyzed using ImageJ.

#### RNAseq cell collection, sequencing, and analysis

Cells were harvested on PID23 in triplicate, and total RNA was isolated using the RNeasy Plus Micro Kit (Qiagen) according to the manufacturer’s instructions. Purified RNA samples were submitted to AmpSeq LLC for library preparation and sequencing. Libraries were sequenced on an Illumina NovaSeq 6000 platform to a depth of 20 million reads per sample. Raw sequencing reads were processed using fastp (v0.23.1) for adapter trimming and quality filtering. Read quality was assessed using FastQC (http://www.bioinformatics.babraham.ac.uk/projects/fastqc/) (v0.11.9) and MultiQC (v1.11) (Ewels et al., 2016). Trimmed reads were aligned to the human reference genome (GRCh38/hg38) using STAR (v2.7.11) (Dobin & Gingeras, 2015). Transcript abundance estimates were generated using Salmon (v1.10.2) (Patro et al., 2017), and summarized to gene-level counts using tximport (v1.30.0) (Soneson et al., 2015). Gene count matrices were imported into R and analyzed using DESeq2 (Love et al., 2014). Genes with low counts (fewer than 10 reads in at least three samples) were filtered prior to differential expression analysis. Counts were normalized using the DESeq2 median-of-ratios method, and dispersion estimates were calculated using the default DESeq2 pipeline. Pairwise comparisons between conditions were then performed to identify differentially expressed genes (DEGs). Adjusted *P* values were calculated using the Benjamini-Hochberg method and log2 fold-change values were shrunk using the apeglm method. Heatmaps were generated using ComplexHeatmap (Gu et al., 2016) in R. Gene function annotation and pathway analysis of DEGs based on differential expression thresholds (adj. *P* value and log2 fold-change as defined for each analysis) was performed using Enrichr (Chen et al., 2013; Kuleshov et al., 2016).

#### Quantification and statistical analysis

Immunostaining quantification was performed manually in ImageJ. For quantification of neuronal markers MAP2, NCAM (Figure 3B): three fields of view (FOV) containing approximately 30 miNs were included for both healthy control and patient-derived miNs. For TUNEL quantification (Figure 3D and 5D): three FOV per coverslip across two coverslips, representing independent reprogrammed cell populations, per condition (total N=6) were included for analysis. All data are represented as mean ± standard error mean (SEM) with dots indicating the percent of DAPI positive cells positive for the marker. Neurite phenotype quantification included two FOV per coverslip across two coverslips (total N=4) per condition (Figure 3C and 5C) and ten FOV per coverslip across two coverslips (total N=20) per condition (Figure 6C). Within each FOV the total number of neurites blebs and broken neurites were manually counted using the cell counter function in Image J. All data are represented as mean ± SEM with dots indicating the number of blebs/broken neurites divided by the number of DAPI positive cells within the FOV.

Statistical significance for the cellular phenotypes was determined using unpaired T-Tests with Welch’s correction in GraphPad Prism (Version 11.0.0) with *P*-values <0.05 considered significant.

#### Statistics and data analysis

Statistical analyses were performed by using GraphPad Prism (version 9). Normality and lognormality test was used to check if the data set has a normal distribution. If not Mann-Whitney test was used to compare the difference between two groups. Kruskal-Wallis test followed by Dunn’s multiple comparison test was used to compare the difference among more than two groups. A P-value <0.05 was considered statistically significant. *** and **** indicate *p* values < 0.001 and < 0.0001, respectively. Statistical significance for the cellular phenotypes was determined using unpaired T-Tests with Welch’s correction in GraphPad Prism (Version 11.0.0) with *P*-values <0.05 considered significant.

## Supplementary Material

1
