# Supplementary material for: Cross-species functional analysis of a de novo DCLK1 variant associated with a neurodevelopmental disorder
Source: Res Sq. 2026 Jul 23:rs.3.rs-10425123. Preprint. [Version 1] doi: 10.21203/rs.3.rs-10425123/v1 (PMC13419596; doi:10.21203/rs.3.rs-10425123/v1)
Supplement: 1 [file NIHPPRS10425123V1-supplement-1.pdf]

SUPPLEMENTARY INFORMATION

Cross-species functional analysis of a *de novo DCLK1* variant associated with neurodevelopmental regression pathology

(Butler et al.)

Table of contents

1. Supplemental Table S1 - Supplemental Table S1: Variant analysis (page 2)
2. Supplemental Table S2 - *C. elegans* strains (page 3)
3. Supplemental Table S3: CRISPR reagents and PCR primers (page 4)
4. Supplemental Figure S1: Longitudinal brain MRI comparison (page 5)
5. Supplemental Figure S2: Developmental regression timeline (page 6)
6. Supplemental Figure S3. Overexpression experiments in *Drosophila* do not reveal major functional impact of the p.P623R variant in SFPQ (page 7)
7. Supplemental Figure S4. Generation of *zyg-8(S382L)* variant and deletion alleles via CRISPR-Cas9 genome editing (page 8)
8. Supplemental Figure S5. Pathway analysis of downregulated genes in patient-derived neurons (PDNs) versus healthy control (HC) neurons (page 9)
9. Undiagnosed Diseases Network Consortium Author List (pages 10-11)

Supplementary Table S1. Variant analysis

	DCLK1	***SFPQ***	***CACNA1A***	***PHKA1***
**Inheritance pattern**				
Zygosity	heterozygous	heterozygous	heterozygous	heterozygous
Inheritance	*de novo*	*de novo*	inherited from father	inherited from mother
**Location and change**				
hg19/GRCh37	chr13:36686046	chr1:35652720	chr19:13345816	X:71872464
hg38/GRCh38	chr13:36111909	chr1:35187119	chr19:13235002	X:72652614
Reference/variant	G/A	G/C	T/C	C/T
Canonical Transcript	NM_001330071.2	NM_005066.3	NM_001127222.2	NM_002637.4
mRNA change	c.683C>T	c.1868C>G	c.5168A>G	c.1175G>A
Amino acid change	p.Ser228Leu	p.Pro623Arg	p.Asp1723Gly	p.Arg392Gln
Consequence	missense variant	missense variant	missense variant	missense variant
**Pathogenicity predictions**				
SIFT score	deleterious (0.0)	deleterious (0.04)	deleterious (0.0)	deleterious (0.0)
Polyphen score	possibly damaging (0.871)	probably damaging (0.94)	probably damaging (0.975)	probably damaging (0.991)
REVEL score	0.697	0.552	0.577	0.939
Alpha missense score	likely pathogenic (0.7968)	likely pathogenic (0.7452)	likely benign (0.1184)	likely pathogenic (0.9509)
CADD score	30	27.6	26.6	26.8
**gnomAD v4.1.1**				
pLI	1	1	1	0
pLI - o/e	0.19 (0.13 - 0.28)	0.15 (0.09 - 0.25)	0.22 (0.18 - 0.27)	0.54 (0.44 - 0.68)
Missense Z score	4.87	1.98	7.41	1.97
Missense Z score - o/e	0.63 (0.59 - 0.67)	0.85 (0.81 - 0.9)	0.71 (0.69 - 0.73)	0.85 (0.8 - 0.9)
Seen in gnomad? v4.1.0	1 time, none in homozygotes	not seen	not seen	1 time, once in hemizygotes

Supplemental Table S2. *C. elegans* strains

Strain	Genotype
VC2010	*C. elegans wild-type parental strain*
SK4005	*zdIs5 [Pmec-4::GFP]*
UDN100587	*zyg-8(udn285) S382S, line #1*
UDN100589	*zyg-8(udn287) S382L, line #1*
UDN100590	*zyg-8(udn288) S382L, line #2*
UDN100592	*zyg-8(udn290) deletion/qC1, line #1*
UDN100630	*zyg-8(udn285) S382S line#1; zdIs5 [Pmec-4::GFP]*
UDN100631	*zyg-8(udn287) S382L line#1; zdIs5 [Pmec-4::GFP]*
UDN100632	*zyg-8(udn290) deletion/qC1 line#1; zdIs5 [Pmec-4::GFP]*

Supplemental Table S3. CRISPR reagents and PCR primers

Name	Sequence (5' to 3')	Description
zyg-8. S382_gRNA	TTTGTCGTGAAGCTCGATTC	Guide RNA near Serine 382
zyg-8. S382_Variant reptemp	ttccagATCCTTCGACCAAGTGCTCCGTG ACCTGACATTcGTaGTcAAGCTCGATctc GGcGCcATTCGAAAACTATTCACACTAT CCGGACGGCCTGTT	Variant (S382L) repair template for udn589
zyg-8. S382_Control reptemp	ttccagATCCTTCGACCAAGTGCTCCGTG ACCTGACATTcGTaGTcAAGCTCGATTC TGGcGCcATTCGAAAACTATTCACACTA TCCGGACGGCCTGTT	Control (S382S) repair template for udn587
zyg-8. Del_gRNA1	tcgaacgATGCCACAAACGT CTT	Guide RNA near 5'UTR
zyg-8. Del_gRNA2	agtcaatatTCAGCTTTCAC ATT	Guide RNA near 3'UTR
zyg-8. Del_repair	atcgctaatttggtgtttttttcggtgttagactatctctactcg aacgatattgactattgactgatatacgcaattaaatatatt cgatatcaattt	Deletion repair template for udn290
zyg-8. S382_F1	ctgattggtcgcagtggg	Genotyping forward primer for S382S and S382L
zyg-8. S382_R1	CATATTCGACGACGTTCCCG	Genotyping reverse primer for S382S and S382L
zyg-8. Del_F1	ctcatctcccgcatttttgg	Genotyping forward primer for deletion
zyg-8. Del_R1	aaataagctgctgcacaattgc	Genotyping reverse primer for deletion
zyg-8. Del_WT_F1	tcaggggcaaatttggattgtc	Genotyping middle primer for WT

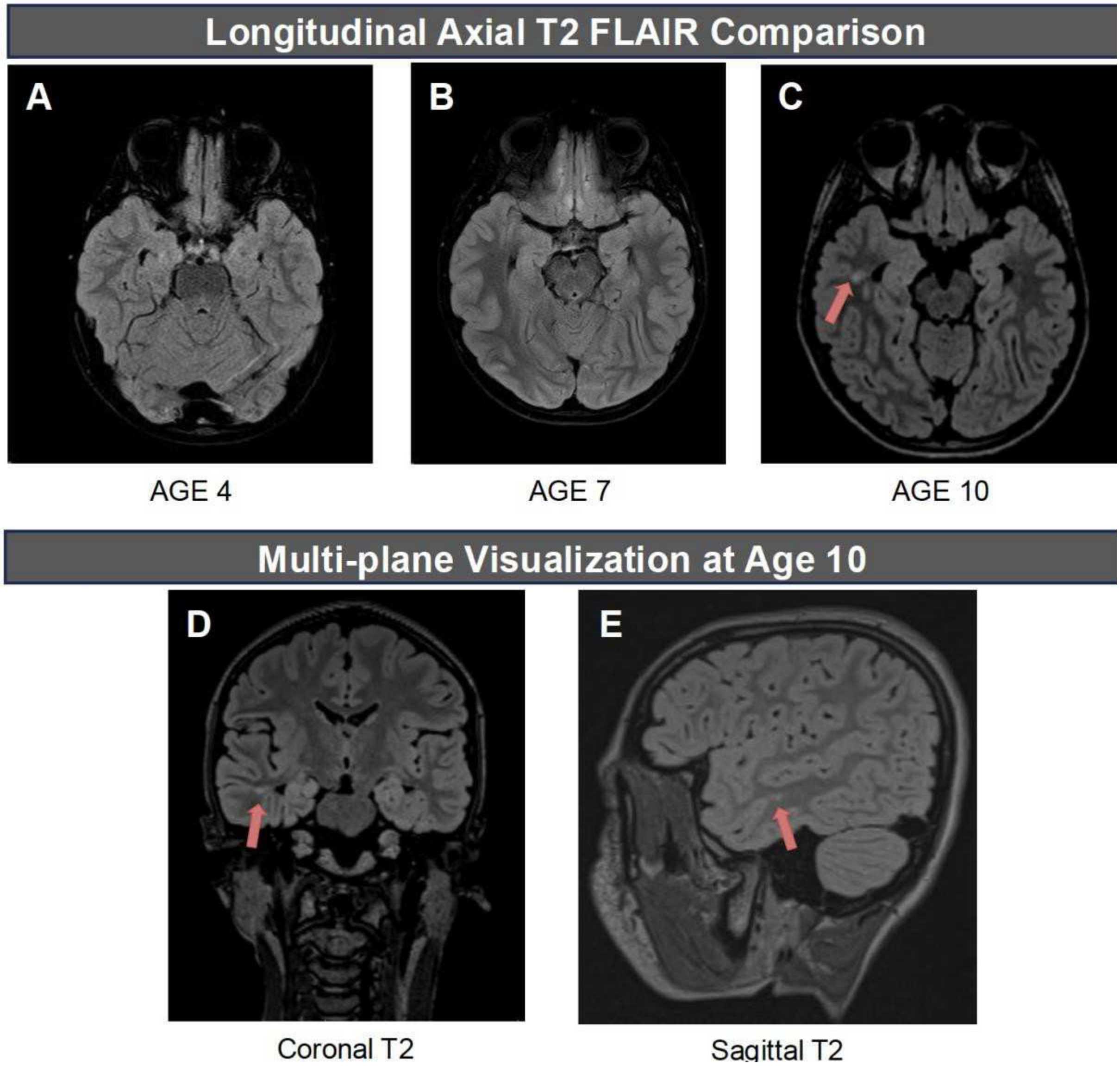

Supplemental Figure S1. Longitudinal brain MRI comparison. Axial T2 FLAIR images at age 4 (A) and age 7 (B) demonstrate no definite abnormality at ages 4 and 7. At age 10 (C), there is a linear focus of T2/FLAIR hyperintensity subjacent to the right middle temporal sulcus extending toward the temporal horn (arrow), with subtle adjacent gray–white matter blurring in the inferior temporal gyrus. Additional coronal (D) and sagittal (E) images at age 10 further localize this finding within the right temporal lobe. The appearance is suggestive of focal cortical dysplasia, with nonspecific gliosis in the differential diagnosis.

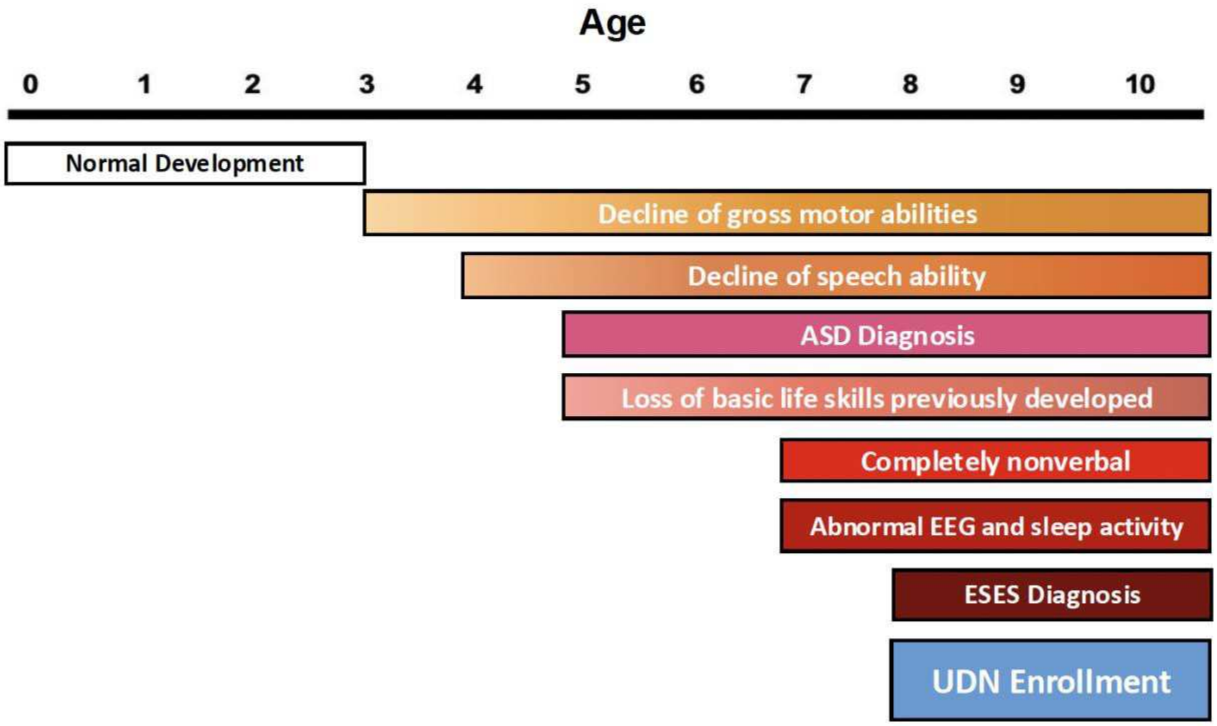

Supplemental Figure S2: Developmental regression timeline. The patient exhibited typical early development with attainment of expected developmental milestones until age 3. At age 3, he experienced a decline in gross motor abilities. Progressive speech regression began at age 4. At age 5, he was diagnosed with autism spectrum disorder (ASD), with continued language deterioration, and additionally demonstrated loss of previously acquired adaptive skills, including inability to dress independently and difficulty operating door locks. By age 7, he had become completely nonverbal and developed abnormal sleep patterns, including insomnia; electroencephalogram (EEG) at that time showed increased spike-wave index during sleep. At age 8, he was diagnosed with epilepsy consistent with electrical status epilepticus in sleep (ESES), characterized by focal impaired awareness seizures. The patient was first evaluated by genetics at age 7 and was enrolled in the Undiagnosed Diseases Network (UDN) by age 8.

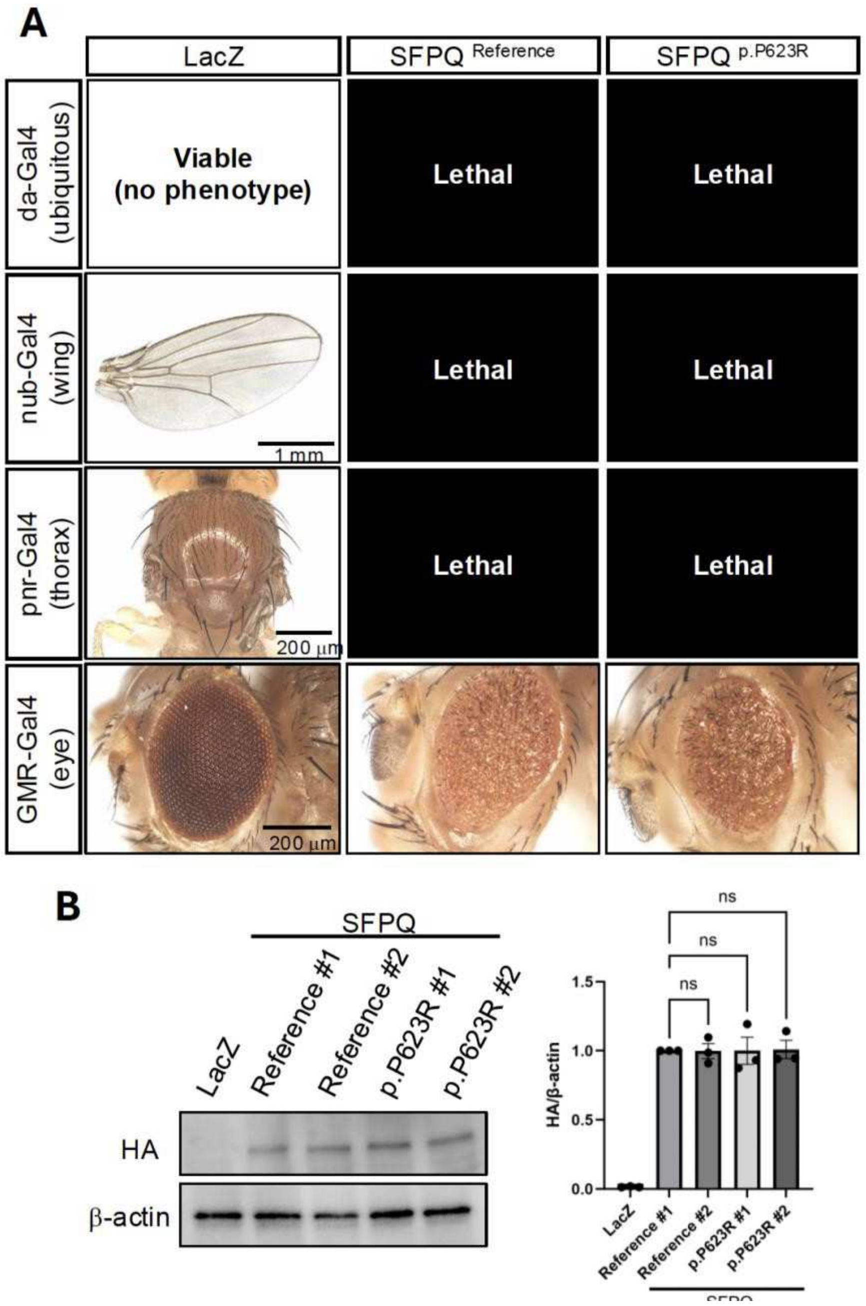

Supplemental Figure S3. Overexpression experiments in *Drosophila* do not reveal major functional impact of the p.P623R variant in SFPQ. (A) Overexpression of both the reference or p.P623R human SFPQ protein using da (ubiquitous), nub(wing), pnr (thorax)-Gal4 and causes lethality, whereas overexpression in the eye (*GMR-Gal4*) causes a rough eye phenotype in both cases. There is no phenotypic difference observed between the reference and the patient variant proteins. (B) Both the reference and p.P623R human SFPQ proteins exhibited similar expression levels based on western blot using two independent transgenic lines. The quantification data of the blots are shown as the mean with SEM.

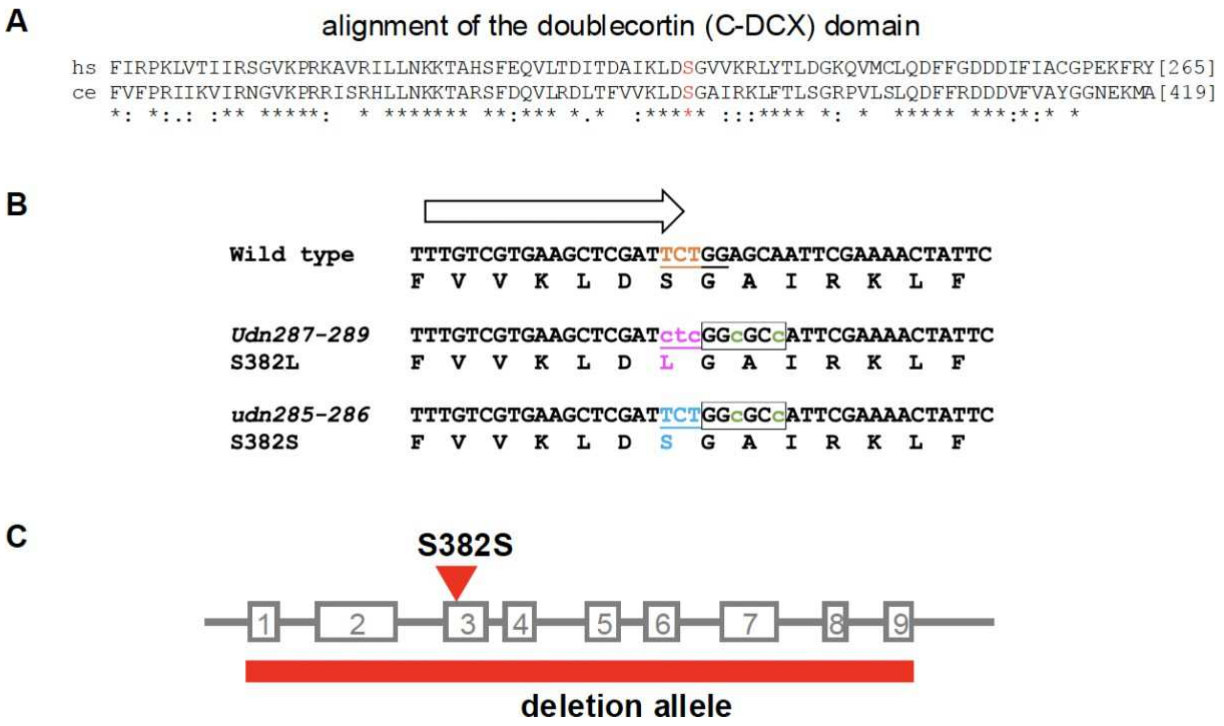

Supplemental Figure S4. Generation of *zyg-8(S382L)* variant and deletion alleles via CRISPR-Cas9 genome editing. (A) Amino acid sequence alignment of the C-terminal DCX domain of human DCLK1 and *C. elegans* ZYG-8. Ser228 in human DCLK1 and the corresponding Ser382 in ZYG-8 are highlighted in red. (B) CRISPR/Cas9-mediated genome editing strategy used to generate *zyg-8(S382L)* alleles. The wild-type sequence encoding Ser382 (TCT) was modified to create the missense allele S382L, in which the serine codon was changed to a leucine codon (CTC). In addition, we introduced synonymous changes to prevent Cas9 re-cutting and to assist in genotyping. For the control allele S382S, TCT codon was unchanged, and only synonymous changes were introduced. Underlined region shows the location of the variant residue. Three independent strains carrying the variant edit (pink) and two independent strains carrying the control edit (blue) were generated for each patient variant. Guide RNA sequence (unfilled arrow). Synonymous changes are in green. Boxed region shows the new restriction enzyme cleavage site introduced to aid genotyping. (C) Schematic representation of the *zyg-8* genomic locus. Exons are shown as numbered boxes. The position of the S382L variant residue within exon 3 is indicated by the red arrowhead. The lower diagram depicts the large deletion allele used in this study, with the deleted genomic region indicated by the red bar.

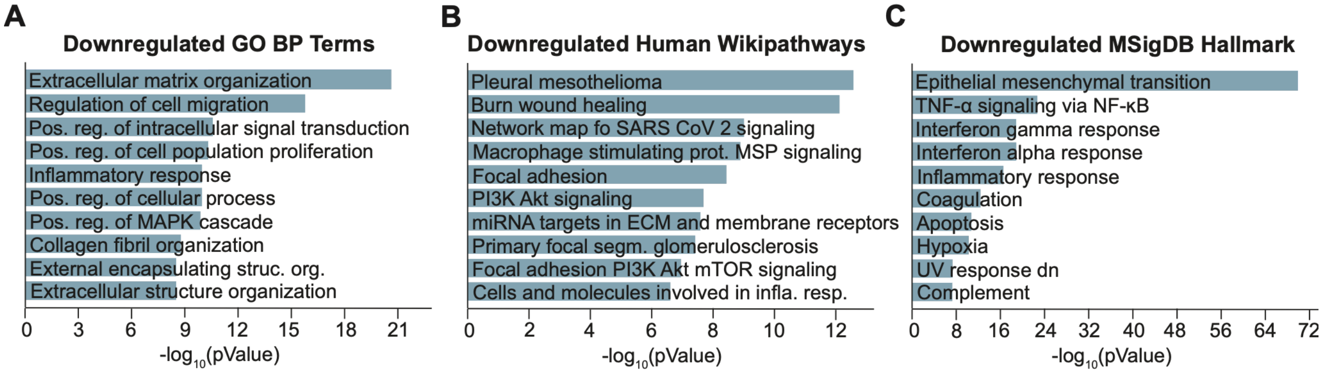

Supplemental Figure S5. Pathway analysis of downregulated genes in patient-derived neurons (PDNs) versus healthy control (HC) neurons. (A) Top downregulated GO BP terms in PDNs. (B) Top downregulated Human Wikipathways in PDNs. (C) Top downregulated MSigDB Hallmark biological processes in PDNs.

Undiagnosed Diseases Network (UDN) Consortium Author list

Jose Abdenur, Maria T. Acosta, David R. Adams, Ben Afzali, Ali Al-Beshri, Eric Allenspach, Raquel L. Alvarez, Justin Alvey, Ashley Andrews, Beatriz Anguiano, Euan A. Ashley, Sanaz Attaripour, Suha Bachir, Carlos A. Bacino, Guney Bademci, Ashok Balasubramanyam, Dustin Baldridge, Erin E. Baldwin, Allen Bale, Elsa Balton, Manisha Balwani, Michael Bamshad, Mafalda Barbosa, Deborah Barbouth, Rebekah Barrick, Donald Basel, Lisa Bastarache, Pinar Bayrak-Toydemir, Taylor Beagle, Alan H. Beggs, Edward Behrens, Megan Bell, Hugo J. Bellen, Paul Berger, Jonathan A. Bernstein, Gerard T. Berry, Louise Bier, Stephanie Bivona, Kirsten Blanco, Lauren Blieden, Elizabeth Blue, Devon Bonner, Brett Bordini, Nicholas Borja, Lorenzo Botto, Steven Boyden, Lauren C. Briere, Elizabeth A. Burke, Lindsay C. Burrage, Manish J. Butte, Russell Butterfield, Peter Byers, William E. Byrd, Kaitlin Callaway, John Carey, George Carvalho, Thomas Cassini, Chun-Hung Chan, Richard Chang, Sirisak Chanprasert, Hsiao-Tuan Chao, Elizabeth C. Chao, Ivan Chinn, Gary D. Clark, Terra R. Coakley, Laurel A. Cobban, Joy D. Cogan, Matthew Coggins, F. Sessions Cole, Erin Conboy, Rosario I. Corona, William J. Craigen, Andrew B. Crouse, Vishnu Cuddapah, Charlotte Cunningham-Rundles, Precilla D'Souza, Hongzheng Dai, Nitsuh K. Dargie, Kahlen Darr, Surendra Dasari, Joie Davis, Margaret Delgado, Esteban C. Dell'Angelica, Nada Derar, Patricia Dickson, Katrina Dipple, Naghmeh Dorrani, Jessica Douglas, Abdul Elkadri, Sara Emami, Lisa T. Emrick, Christine M. Eng, Cecilia Esteves, Rachel Evard, Kimberly Ezell, Layal F. Abi Farraj, Elizabeth L. Fieg, Paul G. Fisher, Brent L. Fogel, Jiayu Fu, William A. Gahl, Eric Gamazon, Rebecca Ganetzky, Eric Gayle, Bruce Gelb, Mark Gerstein, Emily Glanton, Ian Glass, Page C. Goddard, Joanna M. Gonzalez, John E. Gorzynski, Brett H. Graham, Andrea Gropman, Meghan C. Halley, Winston Halstead, Rizwan Hamid, Neil Hanchard, Kelly Hassey, Caroline Hendry, Frances High, Fuki M. Hisama, Ingrid A. Holm, Jason Hom, Martha Horike-Pyne, Alden Huang, Yan Huang, Monika Weisz Hubshman, Anna Hurst, John A. Phillips III, Wendy Introne, Ayuko Iverson, Gail P. Jarvik, Orpa Jean-Marie, Lauren Jeffries, Joanna Jen, Tanner D Jensen, Yong-Hui Jiang, Vaidehi Jobanputra, Oguz Kanca, Yigit Karasozen, Odelya Kaufman, Laura Keehan, Shamika Ketkar, Dana Kiley, Gonench Kilich, Eric Klee, Shilpa N. Kobren, Isaac S. Kohane, Jennefer N. Kohler, Bruce Korf, Susan Korrick, Mary Koziura, Elijah Kravets, Alyson Krokosky, Runjun Kumar, Seema R. Lalani, Brendan C. Lanpher, Ian R. Lanza, Kumarie Latchman, Kimberly LeBlanc, Brendan H. Lee, Miranda Leitheiser, Monkol Lek, Kathleen A. Leppig, Mia Levanto, Richard A. Lewis, Rachel Li, Khurram Liaqat, Pengfei Liu, Nicola Longo, Joseph Loscalzo, Richard L. Maas, Calum A. MacRae, Ellen F. Macnamara, Valerie V. Maduro, Rachel Mahoney, MayChristine V. Malicdan, Tarun KK Mamidi, Shrikant Mane, Lili Mantcheva,

Rong Mao, Ronit Marom, Gabor Marth, Beth A. Martin, Martin G. Martin, Julian A. Martínez-Agosto, Shruti Marwaha, Taylor Maurer, Julie McCarrier, Allyn McConkie-Rosell, Ashley McMinn, Erin McRoy, Hector Rodrigo Mendez, Matthew Might, Mohamad Mikati, Danny Miller, Alexander Miller, Ghayda Mirzaa, Breanna Mitchell, Stephen B Montgomery, Paolo Moretti, Jennifer Morgan, Marie Morimoto, Tahseen Mozaffar, John J. Mulvihill, Lindsay Mulvihill, Michael Muriello, Sandesh Nagamani, Mariko Nakano-Okuno, Thomas J. Nicholas, Donna Novacic, Devin Oglesbee, Carol Oladele, James P. Orengo, Rebecca Overbury, Laura Pace, Stephen C. Pak, J. Carl Pallais, Neil H. Parker, Alex Paul, LéShon Peart, Lakshitha Perera, Seth Perlman, Leoyklang Petcharet, Jennifer E. Posey, Lorraine Potocki, Rakale C. Quarells, Aaron Quinlan, Daniel J. Rader, Ramakrishnan Rajagopalan, Deepak A. Rao, Anna Raper, Wendy Raskind, Adriana Rebelo, Chloe M. Reuter, Lynette Rives, Lance H. Rodan, Martin Rodriguez, María José Ortuño Romero, Jill A. Rosenfeld, Elisabeth Rosenthal, Francis Rossignol, Bianca E. Russell, Marla Sabaii, Mohamad Saifeddine, Jacinda B. Sampson, Suzanne Sandmeyer, Timothy Schedl, Jason Schend, Lisa Schimmenti, Kelly Schoch, Jennifer Schymick, Daryl A. Scott, Teodoro Jerves Serrano, Elaine Seto, Mariya Shadrina, Vandana Shashi, Emily Shelkowitz, Jimann Shin, Susan Shin, Saskia Shuman, Cathy Shyr, Edwin K. Silverman, Giorgio Sirugo, Kathy Sisco, Tammi Skelton, Cara Skraban, Carson A. Smith, Kevin S. Smith, Jared Sninsky, Lilianna Solnica-Krezel, Ben Solomon, Albert R. La Spada, Michele Spencer-Manzon, Rebecca C. Spillmann, Maija-Rikka Steenari, Andrew Stergachis, Joan M. Stoler, Kathleen Sullivan, Shamil R. Sunyaev, David A. Sweetser, Barbara N. Pusey Swerdzewski, Virginia Sybert, Holly K. Tabor, Queenie Tan, Arjun Tarakad, Herman Taylor, Mustafa Tekin, Willa Thorson, Cynthia J. Tifft, Camilo Toro, Alyssa A. Tran, Kayla M. Treat, Brianna Tucker, Rachel A. Ungar, Filippo Pinto e Vairo, Adeline Vanderver, Andres Vargas, Vasilis Vasiliou, Francisco Bustos Velasquez, Matt Velinder, James Verbsky, Francesco Vetrini, Eric Vilain, Dave Viskochil, Tiphanie P. Vogel, Colleen E. Wahl, Melissa Walker, Nicole M. Walley, Jennifer Wambach, Emily Wang, Michael F. Wangler, Isum Ward, Patricia A. Ward, Alistair Ward, Stephanie M. Ware, Daniel Wegner, Corrine K. Welt, Mark Wener, Monte Westerfield, Matthew T. Wheeler, Jordan Whitlock, Laurens Wiel, Brandon M Wilk, Lynne A. Wolfe, Heidi Wood, Kim Worley, Elizabeth A Worthey, Changrui Xiao, Hua Xu, Shinya Yamamoto, Hui Zhang, Michael Zimmermann, Stephan Zuchner
